# Pink1-mediated mitophagy in the endothelium releases proteins encoded by mitochondrial DNA and activates neutrophil responses during inflammation

**DOI:** 10.7554/eLife.82205

**Published:** 2026-07-01

**Authors:** Priyanka Gajwani, Li Wang, Koushik Debnath, Pierina Danos, Young-Mee Kim, Shubhi Srivastava, Zijing Ye, Sarah Krantz, Dong-Mei Wang, Chinnaswamy Tiruppathi, Peter T Toth, Sriram Ravindran, Jalees Rehman

**Affiliations:** 1 Department of Biochemistry and Molecular Genetics, University of Illinois, College of Medicine Chicago United States; 2 Department of Oral Biology, University of Illinois, College of Dentistry Chicago United States; 3 University of Illinois Cancer Center Chicago United States; 4 Department of Pharmacology and Regenerative Medicine, University of Illinois, College of Medicine Chicago United States; 5 Research Resources Center, University of Illinois Chicago Chicago United States; The University of Tokyo Japan; Yale University United States

**Keywords:** mitophagy, mitochondria, inflammation, neutrophil, formylated proteins, endothelial cells, Human, Mouse

## Abstract

Eukaryotic mitochondria are characterized by several features that represent vestiges of their prokaryotic ancestry. One such feature is the N-terminal formylation of proteins encoded by mitochondrial DNA that undergo translation by mitochondrial ribosomes. N-formylated proteins are also released by bacteria and trigger activation of immune cells, such as neutrophils. Growing evidence indicates that circulating levels of mitochondrial formyl proteins are elevated in the serum of patients with excessive inflammatory responses. However, the mechanisms by which they are released into circulation are not known. In this study, we have identified vascular endothelial cells as a source of Pink1-dependent release of mitochondrial formyl proteins in response to inflammatory mediators. Mechanistically, the mitophagy mediator Pink1 is stabilized by inflammatory activation of endothelial cells, promoting mitophagy and mitochondrial formyl peptide release both in mice and primary human endothelial cells. Using nanoparticle delivery of *Pink1*-targeting sgRNA in mice expressing endothelial-specific Cas9, we developed a mouse model in which *Pink1* is specifically depleted in the endothelium. Deletion of endothelial *Pink1* decreased circulating formyl peptide levels, lowered lung neutrophil infiltration, and reduced mortality in mice. We thus propose that endothelial cells upregulate pro-inflammatory mitophagy in response to inflammation, leading to the release of mitochondrial formyl peptides and detrimental neutrophil recruitment into the lung.

## Introduction

Vascular endothelial cells lining the blood vessels are the first point of contact for circulating immune cells that transmigrate into a tissue and therefore, endothelial cells play a critical role in regulating immune response (Kolaczkowska and Kubes, 2013; Amersfoort et al., 2022). Through modulation of vascular permeability, expression of surface markers, and secretion of signaling factors, endothelial cells recruit and direct immune cells such as neutrophils to infected tissues (Pober and Sessa, 2007; Muller, 2016; Al-Soudi et al., 2017; Filippi, 2019). Thus, endothelial function is an important determinant of the rate and extent of inflammatory activation. Recent studies of endothelial function suggest a critical role of endothelial metabolism in driving endothelial migration and angiogenesis (De Bock et al., 2013a; De Bock et al., 2013b). However, beyond ATP production, endothelial mitochondria serve as important signaling organelles, through the control of ROS, NO, and Ca^2+^ signaling (Quintero et al., 2006; Kluge et al., 2013; Tiku et al., 2020). Endothelial mitochondria undergo depolarization in response to inflammatory mediators such as the cytokine TNFα (Chen et al., 1999; Corda et al., 2001), but the underlying molecular mechanisms and impact on host defense have not been defined.

Damaged mitochondria are typically sequestered away from the rest of the mitochondrial pool and targeted to the lysosomes for degradation through the process of mitochondrial autophagy, referred to as mitophagy (Onishi et al., 2021). Mitophagy most often occurs through the Pink1/Parkin pathway, which results in the ubiquitination of damaged mitochondria that are then engulfed by an autophagosome and transported to the lysosome (Palikaras et al., 2018; Ng et al., 2021). Thus, mitophagy generally serves as a quality control mechanism to maintain cellular health. Intriguingly, the global deletion of Parkin leads to reduced endothelial inflammatory activation, suggesting a pro-inflammatory role for mitophagy (Letsiou et al., 2017). Several recent studies have suggested that mitochondria and mitochondrial damage-associated molecular patterns (DAMPs) are actively released by cells in response to inflammatory and other stimuli (Zhang et al., 2010; Dorward et al., 2017; D’Acunzo et al., 2021) and can promote pro-inflammatory responses (Puhm et al., 2019).

Given their endosymbiont evolutionary origin, mitochondria contain several remnants of their prokaryotic ancestors, which can trigger immune responses in mammals (Zhang et al., 2010). Proteins encoded by mitochondrial DNA are translated in the mitochondria by ribosomes that resemble prokaryotic translation machinery and differ from their nuclear-encoded counterparts because the initiating methionine contains an additional N-formyl group (Tucker et al., 2011). Bacterial peptides and proteins contain N-formyl groups, which are recognized by mammalian immune cells and initiate inflammatory activation (Bloes et al., 2015). Interestingly, endogenous mitochondrial formylated proteins that are released by mammalian cells can also bind to formyl peptide receptors (FPRs) on the surface of innate immune cells, inducing activation and transmigration similar to what is observed when bacterial formyl peptides bind to the FPRs (Rongvaux, 2018). While this phenomenon has been noted in sterile injury due to physical trauma (McDonald et al., 2010), additional studies suggest that mitochondrial formylated proteins are elevated in the serum of patients with inflammatory diseases such as sepsis, COVID-19, and Acute Respiratory Distress Syndrome (ARDS; Wenceslau et al., 2015; Kwon et al., 2021; Yuan et al., 2021; Kuley et al., 2023; Lu et al., 2025), although it is not known which cell types release these DAMPs and which signaling pathways trigger the release.

In this study, we observed that endothelial cells in the lung upregulate mitophagy in response to the systemic delivery of the bacterial endotoxin lipopolysaccharide (LPS) in vivo. The inflammatory mediator TNFα, which is released by immune cells in response to LPS, activates the mitophagy initiator Pink1. Mice with endothelial-specific depletion of *Pink1* using targeted CRISPR/Cas9 editing exhibited decreased mitophagy, attenuated serum levels of mitochondrial formyl peptides, reduced neutrophil invasion into the lungs, and improved survival to LPS-induced endotoxemia. These data suggest that endothelial Pink1 triggers a pro-inflammatory amplification pathway, which could be targeted to reduce excessive inflammatory responses.

## Results

### Endotoxemic inflammation induces mitophagy in lung vascular endothelial cells

**Figure 1.**
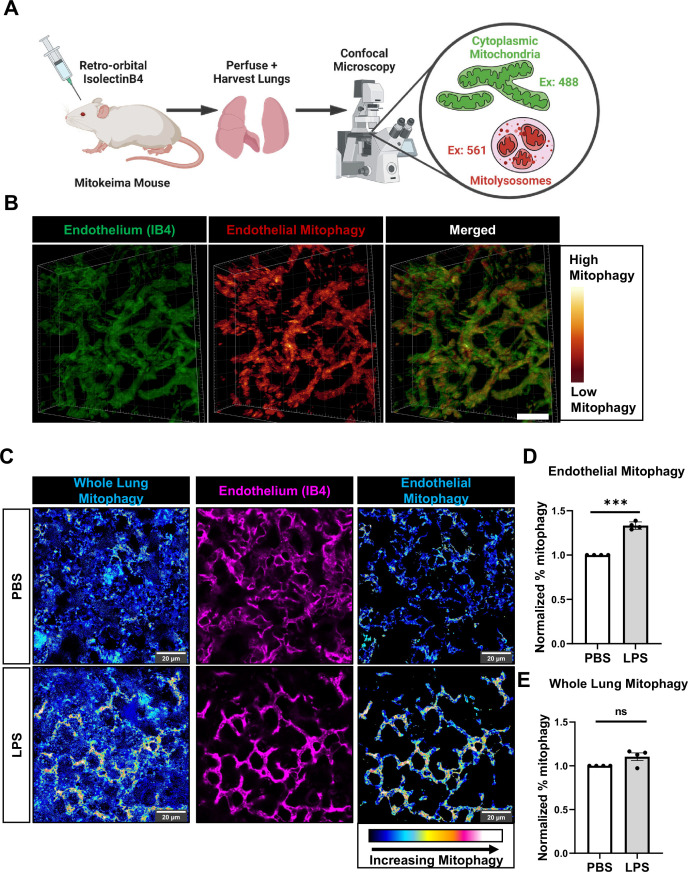
Lung vascular endothelial cells initiate mitophagy in response to endotoxemic inflammation. Mice expressing mitophagy biosensor mitoKeima (mt-Keima) were injected with Isolectin-B4 to label endothelial cells. Lungs were harvested and perfused, and mitophagy was visualized in the whole, un-sectioned lung by confocal microscopy (**A**). This schematic was generated using BioRender.com. A 3D mask of the endothelium was constructed and used to isolate the mt-Keima acidic/neutral ratio specifically in endothelial cells (**B**). Scale Bar: 10 μm. Using this method, mitophagy was measured in an endotoxemia model of inflammation. Lungs from mt-Keima mice were visualized 6 hr post-i.p. Lipopolysaccharide (LPS) injection (8 mg/kg) (**C**). Endothelial (**D**) and whole lung (**E**) mitophagy was measured by calculating the ratio of acidic to neutral mt-Keima (n=4 mice, 10–20 fields of view per mouse). The area of mitolysosomes was quantified as the area of the ratio above threshold. Data represent mean ± SEM. Statistical significance between PBS- and LPS-treated mice was evaluated by t-test. Source data for (**D**) and (**E**) are available in Figure 1—source data 1. Figure 1—source data 1.Spreadsheet containing the original source data for Figure 1D and E.

Given the growing evidence that mitophagy plays an important role in modulating immune and epithelial responses to lung inflammation (Gkikas et al., 2018; Li et al., 2023; Tang et al., 2023), we first sought to determine whether inflammation induced mitophagy in the vasculature in vivo. We evaluated mitophagy using the mitophagy reporter Mitokeima (mt-Keima) mice (Sun et al., 2017). These mice globally express the mitophagy biosensor mt-Keima, which has dual pH-dependent excitation peaks. Thus, mt-Keima distinguishes between cytosolic mitochondria at neutral pH and lysosomal mitochondria, ‘mitolysosomes’, that are at an acidic pH. To label the vascular system, mice were retro-orbitally injected with a fluorescently labeled lectin (DyLight 647-conjugated Isolectin-B4) that specifically binds to mouse endothelial cells. Whole lung lobes were perfused with PBS and imaged immediately after harvesting. Ex vivo imaging by confocal microscopy revealed regions of high and low mitophagy (defined as the acidic:neutral mt-Keima ratio; Figure 1A, Figure 1—figure supplement 1). To distinguish between endothelial and non-endothelial mitophagy, we generated an image ‘mask’ based on the endothelial Isolectin-B4 channel and applied it to the ratio of Acidic:Neutral mt-Keima. A 3D reconstruction of lung vascular mitophagy generated using Imaris image analysis software showed that this method accurately isolates the ratiometric mt-Keima signal from the endothelium, while excluding the surrounding non-endothelial tissue (Figure 1B).

**Figure 1—figure supplement 1.**
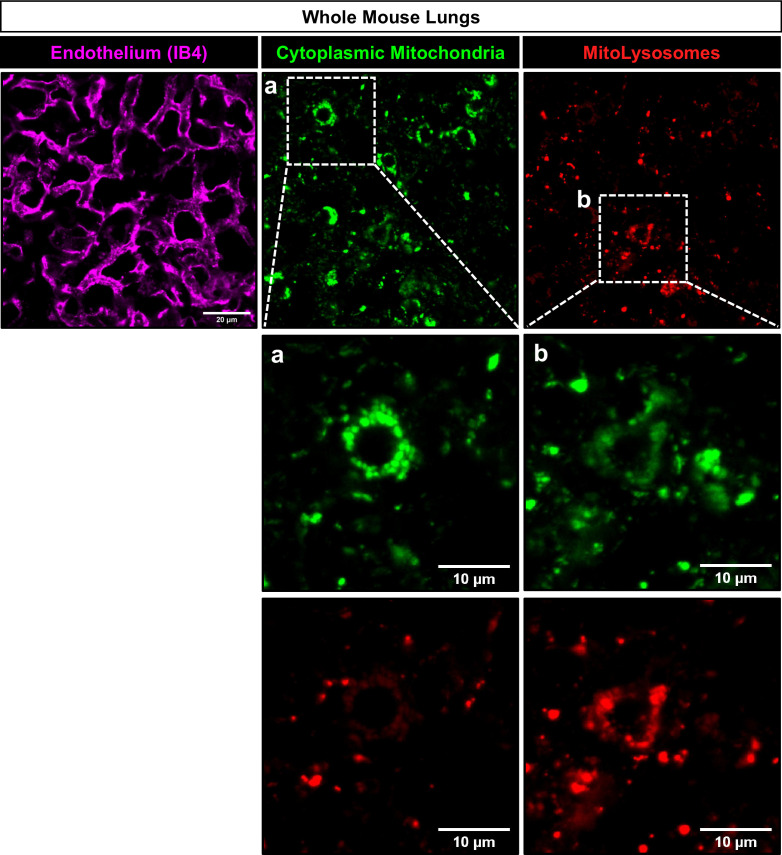
Ex vivo visualization of mt-Keima in the mouse lung. Representative images of lungs from mt-Keima mice injected with Isolectin-B4 (IB4) to visualize the endothelium (magenta). Relative intensities of neutral, cytoplasmic mitochondria (green) and acidic, lysosomal mitochondria (red) are compared to identify regions of lower mitophagy (expanded inset a) and higher mitophagy (expanded inset b).

We applied this method to quantify mitophagy in the lungs of mice under endotoxemia-induced inflammation. The mt-Keima mice were intraperitoneally injected with sub-lethal endotoxin LPS, using PBS as a vehicle control. Lungs were isolated and immediately imaged 6 hr post-LPS injection. Compared to PBS-injected control mice, LPS-injected mice had an approximately 30% increase in endothelial mitophagy (Figure 1C and D). Mitophagy in the whole lung did not significantly change (Figure 1E). Thus, these results indicate that systemic inflammation induced by LPS specifically induces mitophagy in the lung vasculature.

### The inflammatory mediator TNFα stabilizes Pink1 on the mitochondria

**Figure 2.**
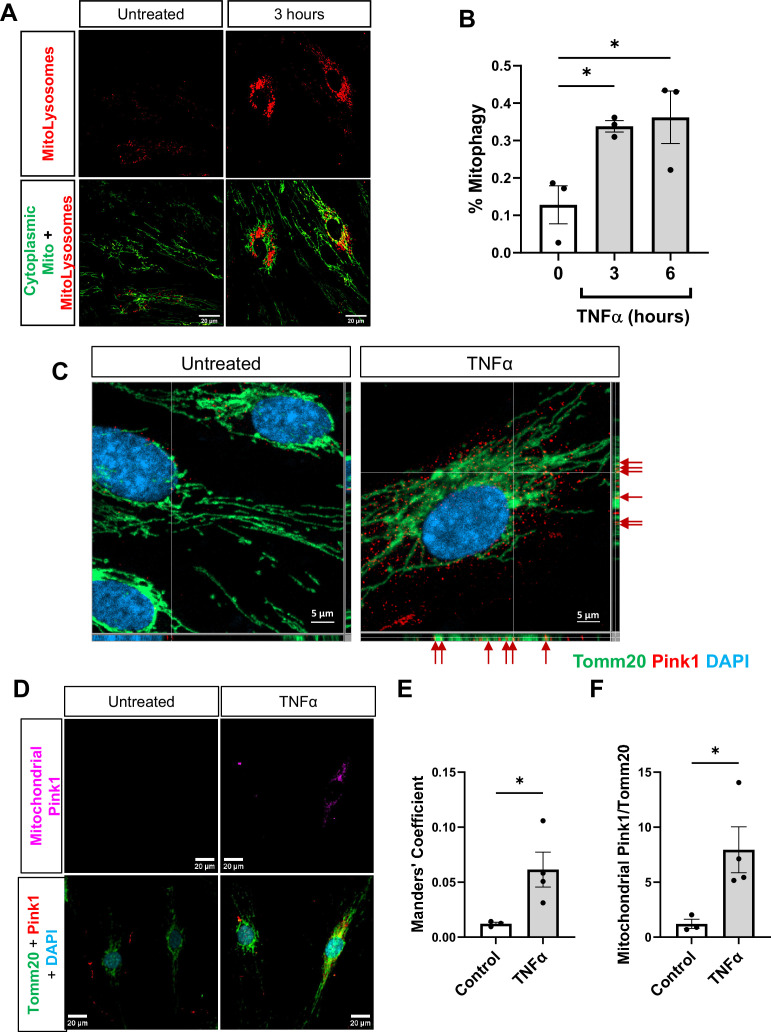
TNFα stabilizes Pink1 on the mitochondria. Human lung microvascular endothelial cells (HLMVECs) expressing mt-Keima were treated with the inflammatory mediator TNFα (10 ng/mL), and mitophagy visualized at 3- and 6 hr post-TNFα exposure, with representative images (**A**). Mitophagy was calculated by measuring the ratio of acidic mitochondria to total mitochondrial area in each visual field (**B**). Data represent mean ± SEM from n=3 independent experiments. Statistical analysis was done using one-way ANOVA. HLMVECs were treated with TNFα for 3 hr, fixed, and immune-stained to visualize Pink1 and the mitochondrial marker Tomm20. Confocal z-stack images were analyzed for Pink1 localization relative to the mitochondria (**C**). Red arrows indicate areas of colocalization of Pink1 with Tomm20. Confocal images (**D**) were analyzed to quantify the Manders’ coefficient of overlap of Pink1 with Tomm20 (**E**), and the total amount of Pink1 in the mitochondrial compartment (**F**). Data represent mean ± SEM. Statistical significance was assessed by Welch’s t-test. Source data for (**B**), (E) and (F) are provided in Figure 2—source data 1. Figure 2—source data 1.Spreadsheet containing the original source data for Figure 2B, E and F.

The inflammatory mediator TNFα is secreted by monocytes in response to infection and LPS-induced inflammation and is a major regulator of inflammatory activation of endothelial cells (Sethi and Hotamisligil, 2021). TNFα induces mitochondrial oxidative stress and fragmentation in endothelial cells, indicating a link to mitochondrial quality control (Forrester et al., 2020). We thus hypothesized that TNFα was the mediator of LPS-induced endothelial mitophagy observed in vivo. Primary human lung microvascular endothelial cells (HLMVECs) were transduced to express mt-Keima and treated with TNFα over a time course of 6 hr to monitor mitophagy by confocal microscopy. Mitophagy was quantified as the ratio of acidic mitochondria/total mitochondria. TNFα significantly induced mitophagy in HLMVECs within 3 hr (Figure 2A and B).

**Figure 2—figure supplement 1.**
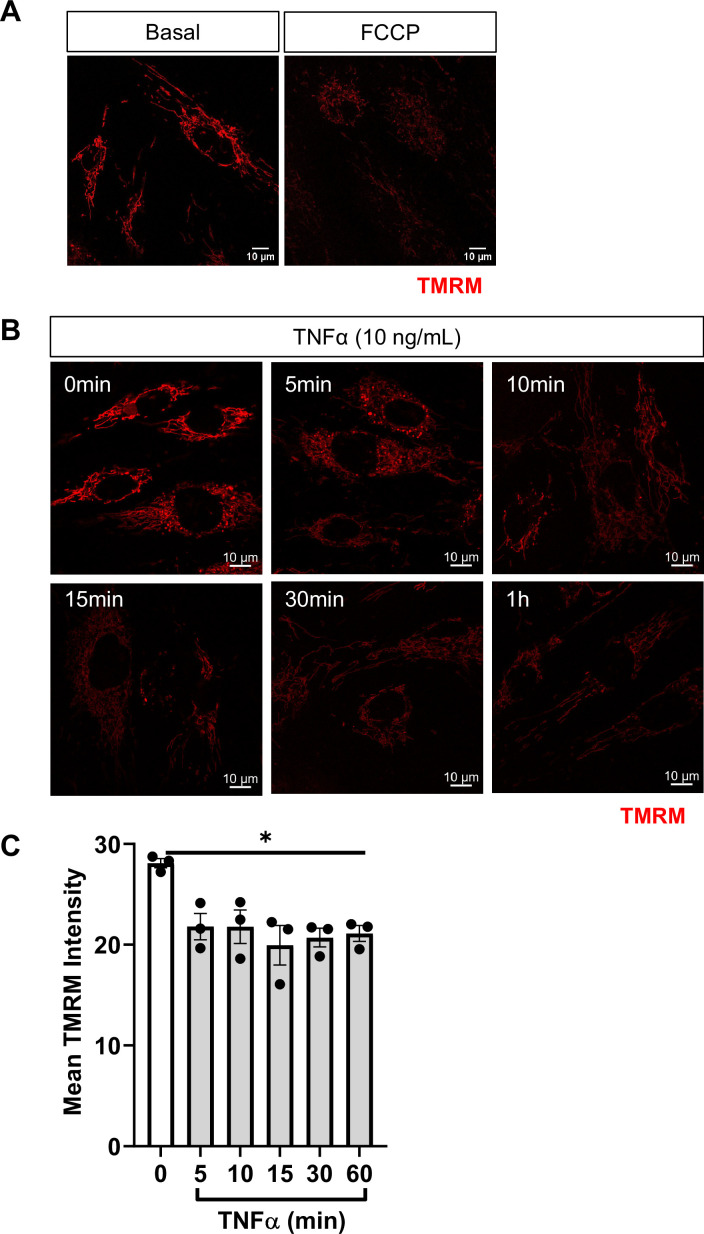
TNFα induces mitochondrial depolarization in endothelial cells. Human lung microvascular endothelial cells (HLMVECs) were treated with TNFα (10 ng/mL) for the indicated times or FCCP (1 µM, 15 min). 30 min prior to imaging, cells were stained with 20 nM tetramethylrhodamine methyl ester (TMRM). Representative images of FCCP-treated HLMVECs (**A**), representative images (**B**), and quantification of the mean TMRM fluorescence intensity ± SEM (**C**) of TNFα-treated HLMVECs from n=3 independent experiments are shown. Statistical analysis was done by one-way ANOVA analysis. Source data for (C) is available in Figure 2—figure supplement 1—source data 1. Figure 2—figure supplement 1—source data 1.Spreadsheet containing the original source data for Figure 2—figure supplement 1C.

**Figure 2—figure supplement 2.**
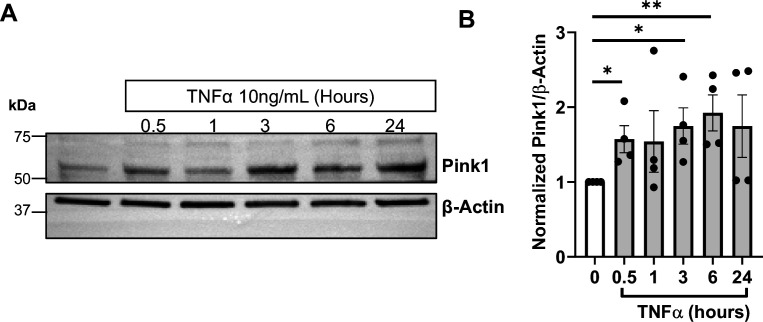
Pink1 protein is stabilized by TNFα. Representative western blot of lysates collected from human lung microvascular endothelial cells (HLMVECs) treated with TNFα for 0.5, 1, 3, 6, and 24 hr (**A**). Quantification of western blots representing mean ± SEM from. n=4 independent experiments (**B**). Statistical analysis was done by t-test. Source Data for (B) are provided in Figure 2—figure supplement 2—source data 1. Uncropped western blot images for (A) are provided in Figure 2—figure supplement 2—source data 2. Figure 2—figure supplement 2—source data 1.Spreadsheet containing the original source data for Figure 2—figure supplement 2B. Figure 2—figure supplement 2—source data 2.Uncropped western blot images for Figure 2—figure supplement 2A, with relevant bands indicated. Figure 2—figure supplement 2—source data 3.Original western blot images for Figure 2—figure supplement 2A.

**Figure 2—figure supplement 3.**
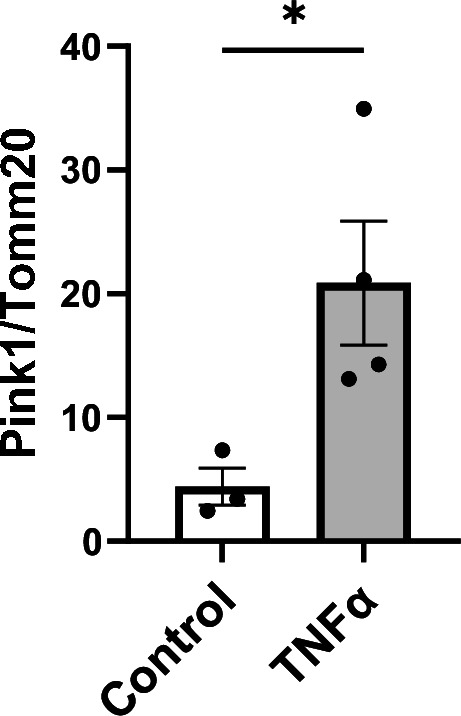
Cellular Pink1 protein levels are increased following treatment with TNFα. Quantification of total Pink1 intensity from confocal images in Figure 2E, normalized to the total intensity of Tomm20 in each image. Data represent mean ± SEM from n=3 independent experiments. Statistical analysis performed by t-test. Source data provided in Figure 2—figure supplement 3—source data 1. Figure 2—figure supplement 3—source data 1.Spreadsheet containing the original source data for Figure 2—figure supplement 3.

We next sought to determine whether the Pink1/Parkin pathway, which is a major mitophagy initiating pathway in multiple cell types (McWilliams and Muqit, 2017), mediated mitophagy in endothelial cells. In healthy, polarized mitochondria, Pink1 is inserted across the mitochondrial membrane, where it is cleaved by mitochondrial proteases and degraded (Matsuda et al., 2010; Yamano and Youle, 2013). However, loss of mitochondrial membrane potential (ΔΨ_m_) prevents this degradation, causing Pink1 to stabilize the outer membrane (Lazarou et al., 2015). We monitored the kinetics of TNFα-induced ΔΨ_m_ changes using the cationic fluorophore tetramethylrhodamine methyl ester (TMRM). When mitochondria are depolarized, such as in response to the mitochondrial uncoupler carbonyl cyanide p‐(trifluoromethoxy)phenylhydrazone (FCCP), they no longer retain TMRM, leading to a dissipation of fluorescence (Figure 2—figure supplement 1A). TNFα induced significant mitochondrial depolarization within 5 min of treatment (Figure 2—figure supplement 1B and C), suggesting mitochondrial depolarization is upstream of mitophagy.

When mitochondria are depolarized, Pink1 is stabilized and recruits the E3 ubiquitin ligase Parkin to ubiquitinate the outer mitochondrial membrane. Parkin-mediated ubiquitination serves as the initiating signal for mitophagy (Lazarou et al., 2015). Thus, stabilization of Pink1 is the first step in the activation of Pink1-mediated mitophagy. HLMVECs treated with TNFα had significantly increased Pink1 protein levels (Figure 2—figure supplement 2A and B). To determine whether Pink1 accumulated in the mitochondria, we co-visualized Pink1 and the mitochondrial membrane protein Tomm20 in TNFα-treated HLMVECs. Consistent with our immunoblotting results, TNFα significantly increased total Pink1 levels (Figure 2—figure supplement 3). Reconstructed z-stack images of the cells showed Pink1 puncta co-localized with Tomm20, which is increased by TNFα (Figure 2C). We quantified the proportion of mitochondria associated with Pink1 by calculating the Manders’ coefficient of colocalization in two-dimensional images of cells. TNFα induced a significant increase in the fraction of Tomm20 that is colocalized with Pink1 (Figure 2D and E), indicating that Pink1 is present on more mitochondria following treatment. To quantify the total amount of Pink1 localized to the mitochondria, we generated a mask of the mitochondrial network and used it to measure mitochondrial Pink1 intensity, normalizing to mitochondrial content as indicated by Tomm20 intensity. Treatment with TNFα induced a 6-fold increase in mitochondrial Pink1 (Figure 2F). These results indicate that TNFα stabilizes Pink1 at the mitochondria.

### Endothelial Pink1 is required for LPS-induced mitophagy

To explore the importance of endothelial Pink1-induced mitophagy in inflammation, we used a CRISPR/Cas9 approach to specifically delete endothelial *Pink1* in vivo. We generated mice that express Cas9 specifically in endothelial cells by crossing knock-in Cas9 mice (Platt et al., 2014) with mice expressing Cre under the endothelial-specific CDH5 (VE-Cadherin) promoter (Alva et al., 2006). *Pink1*-targeting sgRNA was then administered to the mice using lipid-based nanoparticles retro-orbitally or intranasally. C57BL/6 mice injected with *Pink1* sgRNA, or Cas9-VE-Cadherin Cre mice injected with control sgRNA were used as wild type (WT) controls.

Plasmid containing sgRNA against *Pink1* was encapsulated in cationic liposome-a formulation previously demonstrated to deliver genes to the lung endothelium via an intravenous route. Because the lung endothelium is the first microvascular bed encountered by intravenous formulations, this approach serves as an effective strategy for targeted endothelial genetic modification (Liu et al., 2019). Intravenous injection of Pink1 sgRNA-containing liposomes led to deletion of Pink1 specifically in the Cas9 expressing endothelium (*Pink1* EC-KD mice) (Figure 3—figure supplement 1). *Pink1* sgRNA induced an approximately 80% reduction in lung endothelial Pink1 in Cas9-expressing mice compared to age-matched WT control mice (Figure 3—figure supplement 2A and B) without affecting Pink1 levels in non-endothelial cells (Figure 3—figure supplement 2C).

**Figure 3.**
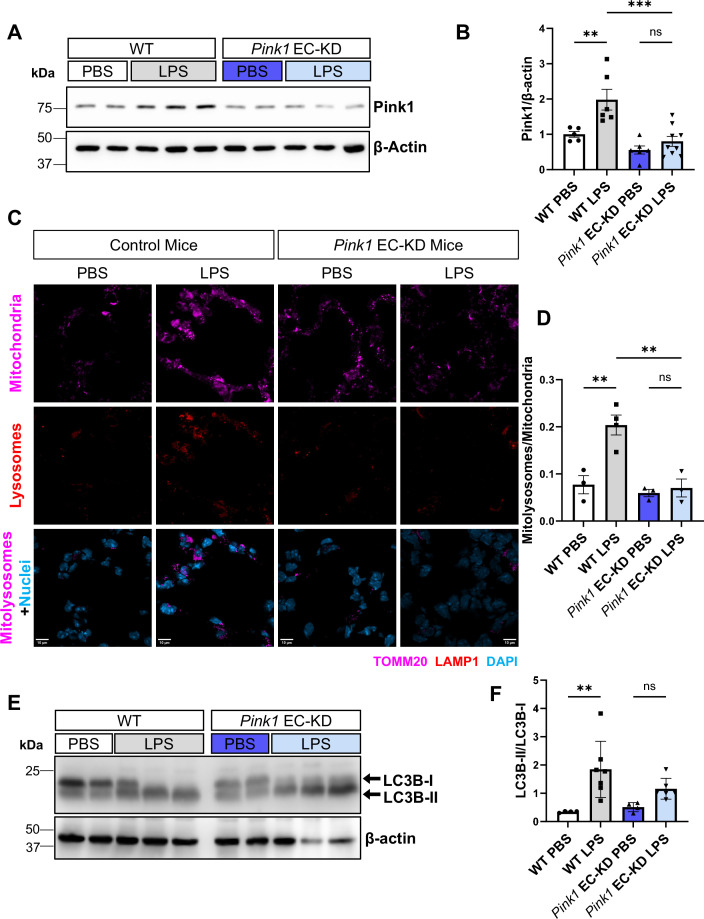
Deletion of endothelial *Pink1* ablates lipopolysaccharide (LPS)-induced mitophagy. Mice were bred to express Cas9 in cells expressing Cre recombinase under a VE-Cadherin promoter, ensuring Cas9 expression specifically in endothelial cells. sgRNA against *Pink1* was delivered to Cas9 expressing, or control C57BL/6 (WT) mice aged 8–12 weeks by retro-orbital i.v. injection of sgRNA containing zwitterionic vesicles. Control and *Pink1* EC-KD mice were subjected to LPS (8 mg/kg body weight, i.p.) or PBS as a control. Lungs were harvested 6 hr post-treatment and analyzed for Pink1 expression via western blot (**A, B**). Mouse lungs were similarly prepared and harvested for immunostaining with the mitochondrial marker Tomm20, and the lysosomal marker Lamp1. DAPI was used to stain the nuclei. The proportion of mitochondria colocalized with lysosomes was calculated by creating a lysosomal mask, quantifying Tomm20 within the masked area, and dividing by total Tomm20 (**C, D**). Conversion of LC3B-I to LC3B-II in lung lysates of mice injected with PBS or LPS for 6 hr was quantified by western blot (**E, F**). Data in (**B**), (**D**), and (**F**) represent mean ± SEM, and statistical significance was evaluated by one-way ANOVA. Source data for (**B**), (**D**), and (**F**) are provided in Figure 3—source data 1. Uncropped western blot images for (A) and (E) are provided in Figure 3—source data 2. Figure 3—source data 1.Spreadsheet containing source data for Figure 3B, D and F. Figure 3—source data 2.Uncropped western blot images for Figure 3A and E, with relevant bands indicated. Figure 3—source data 3.Original western blot images for Figure 3A and E.

Using these mice, we first examined whether LPS stabilizes endothelial Pink1 in vivo. *Pink1* EC-KD and control WT mice were injected with sub-lethal LPS, and lung endothelial cells were isolated 6 hr post-injection. Similar to our observations in TNFα-treated HLMVECs, LPS significantly increased endothelial Pink1 protein levels, suggesting that inflammation also activates Pink1 in vivo (Figure 3A and B). Importantly, in *Pink1* EC-KD mice, Pink1 protein levels remained low even after LPS exposure, signifying that residual levels of Pink1 are not sufficient to compensate for Pink1 deletion by post-translational stabilization.

**Figure 3—figure supplement 1.**
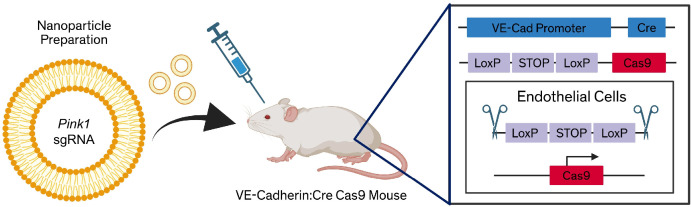
Schematic representation of nanoparticle injection of Pink1 gRNA in Cas9-VECre mice. Generated using BioRender.com.

**Figure 3—figure supplement 2.**
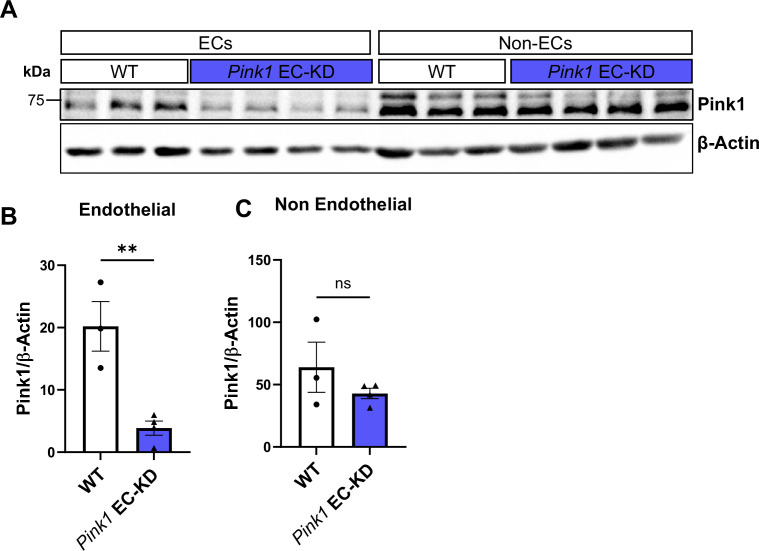
Liposomal delivery of Pink1 sgRNA deletes Pink1 in endothelial cells, but not in non-endothelial cells. sgRNA against Pink1 was delivered to Cas9 expressing, or control C57BL/6 mice aged 8–12 weeks by retro-orbital i.v. injection of sgRNA containing liposomes. Lungs were harvested after 24 hr and evaluated for Pink1 knockdown efficiency by western blot (**A**). Administration of liposomal Pink sgRNA led to an ~80% knockdown in endothelial cells but no significant knockdown in non-endothelial cells (**B, C**). Data represent mean ± SEM from n=3-4 mice per group. Statistical analysis was performed by t-test. Source data for (B) and (C) are provided in Figure 3—figure supplement 2—source data 1. Uncropped western blot images for (A) are available in Figure 3—figure supplement 2—source data 2. Figure 3—figure supplement 2—source data 1.Spreadsheet containing source data for Figure 3—figure supplement 2B and C. Figure 3—figure supplement 2—source data 2.Uncropped western blot images for Figure 3—figure supplement 2A, with relevant bands indicated. Figure 3—figure supplement 2—source data 3.Original western blot images for Figure 3—figure supplement 2A.

Next, we examined whether Pink1 was required for LPS-mediated mitophagy in the lung endothelium. Lungs from *Pink1* EC-KD and control WT mice injected with LPS were sectioned and immuno-stained with the lysosomal marker LAMP1 and the mitochondrial marker Tomm20. The colocalization of LAMP1 and Tomm20 was used to measure mitolysosomes, where mitochondria have been delivered to lysosomes for degradation. Consistent with our observations in mt-Keima mice, 6 hr of LPS treatment increased mitolysosomes (Figure 3C and D). Endothelial deletion of Pink1 abolished LPS-induced mitophagy in the lungs, indicating that Pink1 is required as the initiator of inflammation-induced mitophagy in endothelial cells. LPS also led to increased conversion of the autophagy-related protein LC3-I to the LC3-II form (Figure 3E and F). Conversion of LC3-I to LC3-II is an indicator of increased autophagosome initiation (Gottlieb et al., 2015). Unlike in WT mice, LPS did not significantly increase LC3 conversion in the *Pink1* EC-KD mice, suggesting that Pink1-mediated mitophagy is a significant contributor to autophagic flux in endothelial cells exposed to LPS.

### Endothelial Pink1 exacerbates endotoxemia-induced death through excessive neutrophil recruitment

**Figure 4.**
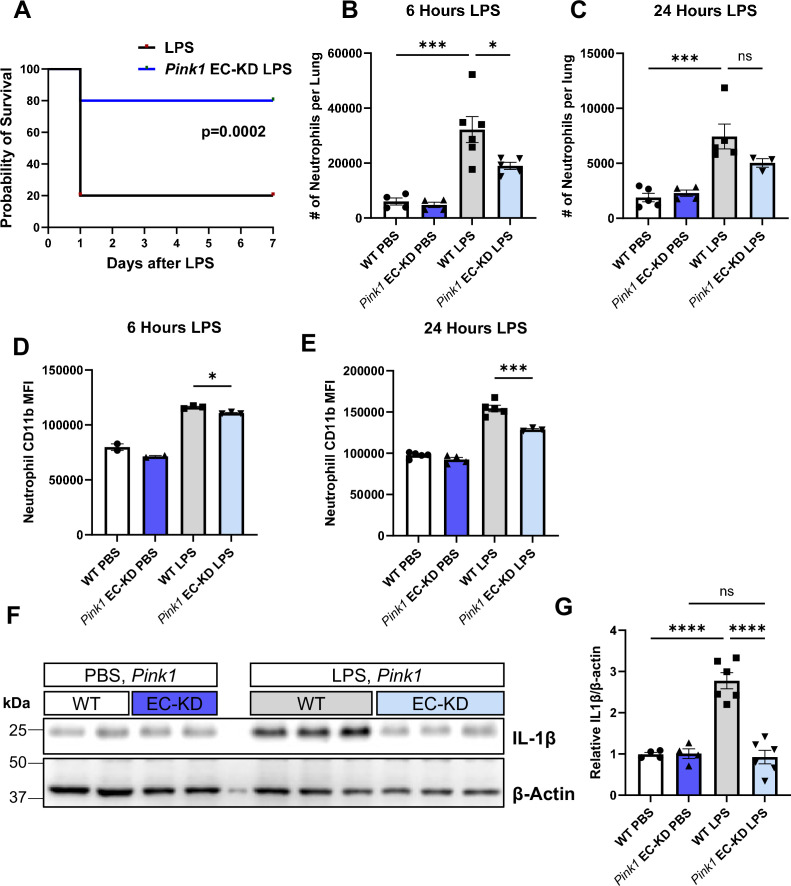
Endothelial Pink1 sensitizes mice to lipopolysaccharide (LPS). Control and *Pink1* EC-KD mice were injected with LPS (10 mg/kg body weight), and survival was monitored over 7 days (**A**). n=20 (10 male and 10 female) mice per group. Statistical analysis was performed using log rank (Mantel-Cox) test. To study neutrophil infiltration and activation, control (WT) and endothelial-specific *Pink1* knockdown (*Pink1* EC-KD) mice were injected with LPS (8 mg/kg body weight). 6- and 24 hr later, lungs were perfused and harvested and analyzed for the number of infiltrated Ly6G+neutrophils by flow cytometry (**B, C**). Data are presented as mean ± SEM. Neutrophil activation was measured by CD11b expression on Ly6G + cells (**D, E**). Data represent median ± SEM from n=3–6 mice per group. IL-1β levels in the whole lung after 6 hr LPS treatment were measured by western blot (**F, G**). n=4 mice for PBS, and n=6 mice for LPS-treated groups, with data represented as mean ± SEM. Statistical significance for (**B–E**) and (**G**) was determined by one-way ANOVA. Source Data for (**B**), (**C**), (**D**), (**E**), and (**F**) are provided in Figure 4—source data 1 . Uncropped western blot images for (**F**) are available in Figure 4—source data 2. Figure 4—source data 1.Spreadsheet containing source data for Figure 4A, B, C, D, E and G. Figure 4—source data 2.Uncropped western blot images for Figure 4F, with relevant bands indicated. Figure 4—source data 3.Original western blot images for Figure 4F.

To determine whether endothelial Pink1 plays a role in determining inflammatory outcome, we first examined the survival of *Pink1* EC-KD mice in response to LPS-endotoxemia. WT and *Pink1* EC-KD mice were intraperitoneally injected with a lethal dose of LPS, and survival was monitored over 7 days. *Pink1* EC-KD mice displayed significantly improved survival compared to WT mice (Figure 4A). This strong pro-survival effect of EC-specific *Pink1* deletion suggests that endothelial Pink1 is a key mediator of pro-inflammatory activation and mortality role in LPS-mediated endotoxemia. Furthermore, the protective effect of *Pink1* EC-KD manifested early on, with most of the WT mice dying on the first day following LPS administration. The early timing of this protective effect suggests that endothelial Pink1 is involved in aggravating inflammatory injury, as opposed to inhibiting pathways involved in lung regeneration. We reasoned that endothelial Pink1 may increase inflammatory injury by increasing LPS-induced vascular permeability. To assess whether *Pink1* EC-KD mice had altered endothelial characteristics, we determined the extent of lung edema, as measured by lung wet-to-dry weight ratio, and vascular permeability as measured by permeability to Evans-Blue-conjugated Albumin (EBA), in LPS injected WT and *Pink1* EC-KD mice. Deletion of endothelial Pink1 did not affect vascular permeability to EBA (Figure 4—figure supplement 1A), nor did it affect loss of the endothelial adherens junction protein VE-Cadherin, which is an important regulator of endothelial barrier function (Figure 4—figure supplement 1B). Additionally, *Pink1* EC-KD did not significantly alter LPS-induced lung edema (Figure 4—figure supplement 2). Thus, although endothelial Pink1 mediates inflammatory lung injury, this is likely not due to direct modulation of the lung vascular barrier integrity.

**Figure 4—figure supplement 1.**
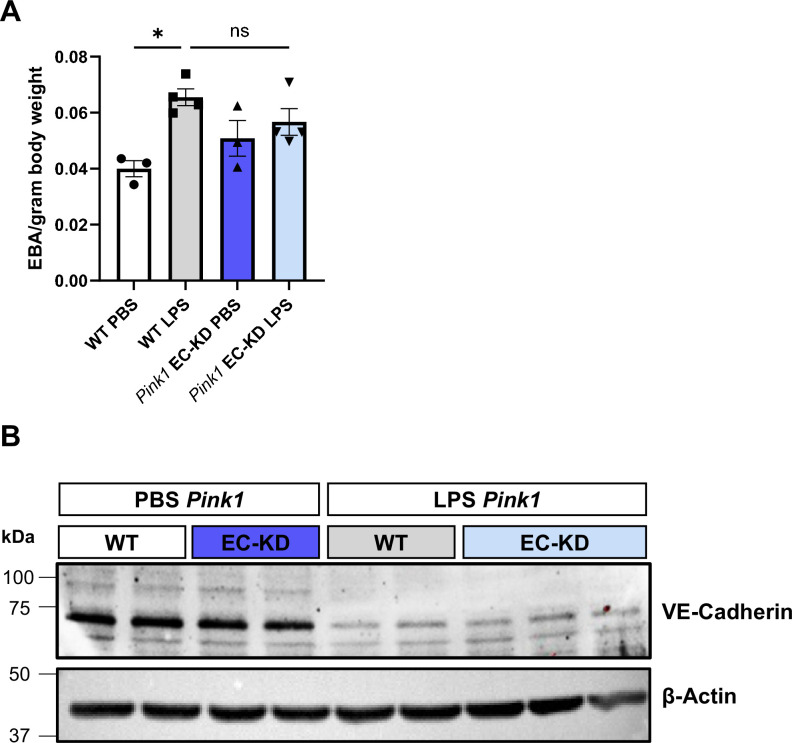
Endothelial Pink1 does not alter lung permeability. Control and *Pink1* EC-KD mice were injected with lipopolysaccharide (LPS; 8 mg/kg), or PBS as a control. Mice were injected with Evans Blue Albumin (EBA), and lungs perfused and harvested 12–14 hr post-injection. EBA in the lungs was quantified (**A**). Data represent mean ± SEM from n=3–4 mice per group. Statistical analysis was performed using one-way ANOVA. VE-Cadherin levels were measured in lungs 6 hr following LPS injection by western blot (**B**). β-Actin was measured as a loading control. n=2–3 mice per group. Source Data for (A) is provided in Figure 4—figure supplement 1—source data 1. Uncropped western blot images for (B) are available in Figure 4—figure supplement 1—source data 2. Figure 4—figure supplement 1—source data 1.Spreadsheet containing source data for Figure 4—figure supplement 1A. Figure 4—figure supplement 1—source data 2.Uncropped western blot images for Figure 4—figure supplement 1B, with relevant bands indicated. Figure 4—figure supplement 1—source data 3.Original western blot images for Figure 4—figure supplement 1B.

**Figure 4—figure supplement 2.**
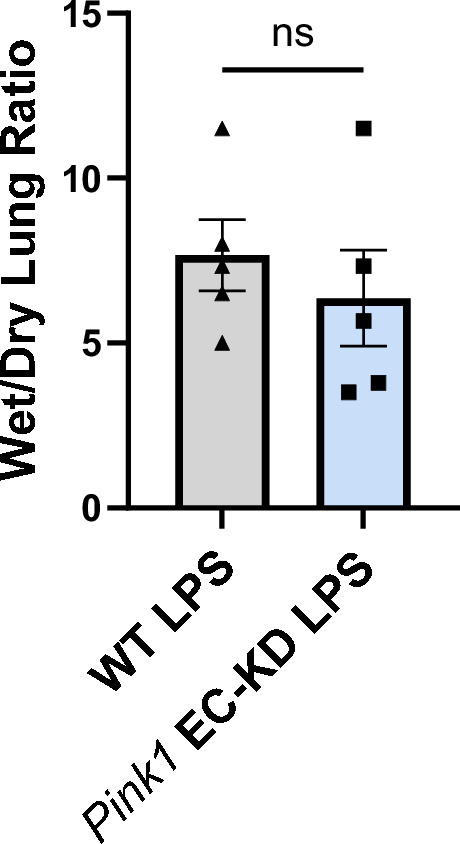
Endothelial Pink1 does not regulate lung edema. Control and *Pink1* EC-KD mice were injected with lipopolysaccharide (LPS; 8 mg/kg), or PBS as a control. 6 hr following lipopolysaccharide (LPS) injection, lungs were harvested and weighed. Following drying at 60 °C, lungs were weighed again, and the wet-to-dry ratio was calculated as a measure of lung edema. Data represent mean ± SEM from n=5 mice per group. Statistical significance was measured by Welch’s t-test. Source data are provided in Figure 4—figure supplement 2—source data 1. Figure 4—figure supplement 2—source data 1.Spreadsheet containing source data for Figure 4—figure supplement 2.

**Figure 4—figure supplement 3.**
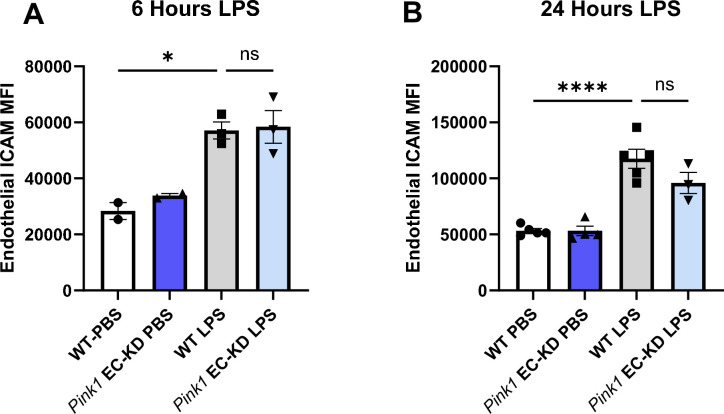
Endothelial Pink1 does not alter endothelial ICAM expression following lipopolysaccharide (LPS) treatment. Control and *Pink1* EC-KD mice were injected with LPS (8 mg/kg). 6- (**A**) and 24- (**B**) hr later, lungs were perfused and harvested and analyzed for ICAM expression in CD31+endothelial cells by flow cytometry. Data are presented as median ± SEM from n=3–5 mice per LPS-treated group. Statistical significance was evaluated by one-way ANOVA. Source data are provided in Figure 4—figure supplement 3—source data 1. Figure 4—figure supplement 3—source data 1.Spreadsheet containing source data for Figure 4—figure supplement 3A, B.

Given the strong protective effect of endothelial *Pink1* deletion in inflammatory injury, and the limited effect on endothelial barrier function, we hypothesized that endothelial Pink1 induces inflammatory injury by acting on the recruitment of immune cells such as neutrophils, which are key mediators of lung injury and death in endotoxemia-induced inflammatory lung injury (Hayashi et al., 2003; Nathan, 2006; Bachmaier et al., 2022). Neutrophils are recruited into the lungs 2–24 hr following systemic delivery of LPS where they are early drivers of inflammation-induced tissue injury (Matute-Bello et al., 2008; Zemans and Matthay, 2017). These kinetics are in line with the observed mitophagy, which occurs within 6 hr of LPS exposure. We thus measured the effect of endothelial Pink1 deletion on LPS-induced infiltration of neutrophils into the lung. WT and *Pink1* EC-KD mice were intraperitoneally injected with sub-lethal LPS, and lungs were harvested 6 and 24 hr post-LPS injections. CD45 and Ly6G were used as markers to label the neutrophil population in whole lung samples. The number of CD45^+^Ly6G^+^ neutrophils was quantified by flow cytometry and normalized to the total lung cell count. Compared to their age-matched controls, *Pink1* EC-KD mice exhibited significantly reduced neutrophil infiltration at 6 hr post-treatment (Figure 4B and C). Interestingly, this difference appeared only at 6 hr but did not persist at 24 hr. Thus, the difference in neutrophil infiltration is concordant with the timing of mitophagy induction. To further establish the importance of endothelial Pink1 in neutrophil-mediated immune response, we measured the activation of neutrophils infiltrated lungs due to LPS-induced inflammation, using CD11b as an activation marker. The increase in membrane CD11b level induced by LPS was dampened in neutrophils from *Pink1* EC-KD mice, showing that neutrophil activation was compromised in *Pink1* EC-KD mice at 24 hr (Figure 4D and E). These results suggest that early neutrophil recruitment is important for effective neutrophil activation, and that both processes are sensitive to *Pink1* EC-KD. The decreased early neutrophil recruitment was also accompanied by significantly reduced levels of the pro-inflammatory cytokine IL-1β in the lung (Figure 4F and G).

One possible mechanism through which endothelial cells may alter neutrophil recruitment is through expression of the adhesion molecule ICAM-1, which is involved in neutrophil adhesion and transmigration into the lung (Yang et al., 2005). Thus, we measured ICAM-1 levels in the CD31^+^ endothelial cells of WT and *Pink1* EC-KD mice injected with LPS for 6 hr. LPS induced similar activation of ICAM-1 in control and *Pink1* EC-KD mice (Figure 4—figure supplement 3), suggesting that changes in neutrophil recruitment are independent of endothelial ICAM-1 expression.

### Endothelial cells release mitochondrial formylated peptides in response to inflammation

**Figure 5.**
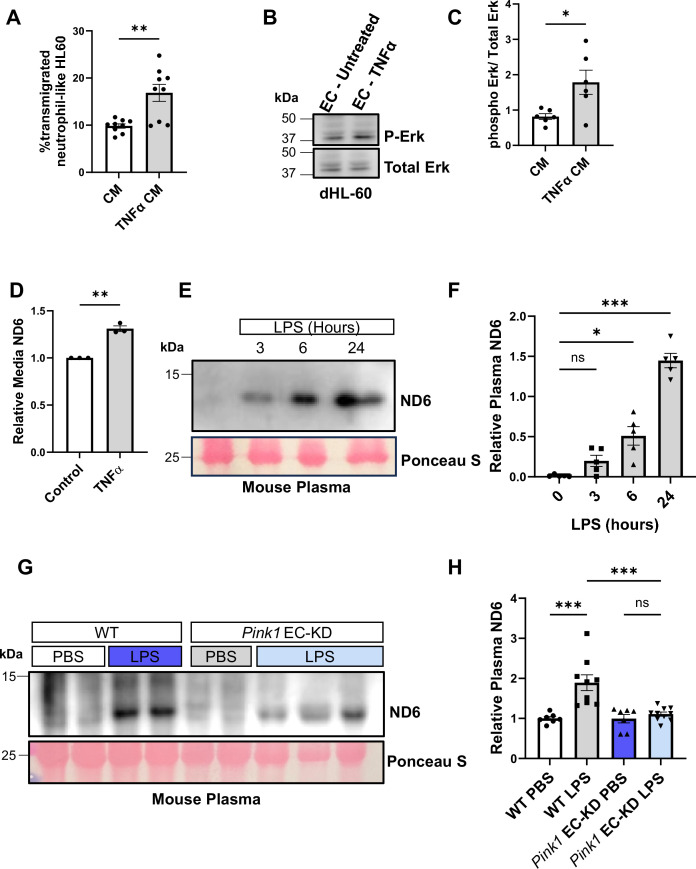
Endothelial cells release ND6 in response to inflammation. Human lung microvascular endothelial cells (HLMVECs) were treated with TNFα for 1 hr, washed, and incubated in low serum media for 24 hr. Conditioned cell culture media was collected and filtered and placed into the lower chamber of a trans-well assay plate. Differentiated HL-60 cells (dHL-60) were placed in the upper chamber. Transmigration in response to media from TNFα-treated and control cells was analyzed (**A**). Data are presented as mean ± SEM from n=3 independent experiments. dHL-60 cells were resuspended in HLMVEC conditioned media, incubated for 10 min, lysed, and analyzed for Erk phosphorylation by western blot (**B, C**). Data represent mean ± SEM from n=3 independent experiments. HLMVECs were treated with TNFα for 24 hr. Conditioned media was then collected and analyzed for the presence of ND6 by ELISA (**D**). Data represent mean ± SEM from n=3 independent experiments. Statistical significance for (**A**), (**C**), and (**D**) was analyzed by t-test. C57BL/6 mice were injected with lipopolysaccharide (LPS; 8 mg/kg, i.p.), and blood plasma collected after 0, 3, 6, and 24 hr. Plasma proteins were precipitated using trichloroacetic acid (TCA, 13%), resuspended in Laemmli buffer, and analyzed by western blot (**E, F**). WT and *Pink1* EC-KD mice were injected with LPS, and plasma collected after 6 hr for TCA precipitation and analysis by western blot (**G, H**). Statistical analysis for graphs (**F**) and (**H**) was performed using one-way ANOVA. Original Data for (**A**), (**C**), (**D**), (**F**), and (**H**) available in , Figure 5—source data 1. Uncropped western blot images for (**B**), (**E**), and (**G**) provided in Figure 5—source data 2. Figure 5—source data 1.Spreadsheet containing source data for Figure 5A, C, D, F and H. Figure 5—source data 2.Uncropped western blot images for Figure 5B, E and G, with relevant bands indicated. Figure 5—source data 3.Original western blot images for Figure 5B, E and G.

We next examined alternative pathways through which endothelial mitochondria may interact with neutrophils. Besides interaction with adhesion molecules, neutrophil recruitment is also heavily regulated by activation of FPRs, which recognize bacterial proteins that contain an additional formyl group on the initiating methionine (Dorward et al., 2015). However, FPRs also drive neutrophil recruitment in ‘sterile’ inflammatory conditions such as LPS-induced inflammation, indicating an endogenous component to FPR activation (Grommes et al., 2014; Yang et al., 2017). Given the endosymbiont origins of mitochondria, proteins encoded in the mitochondrial genome are also N-formylated and can activate the FPR receptors, activating the Erk pathway to drive neutrophil recruitment (Dorward et al., 2015; Zhang et al., 2016; Dorward et al., 2017; Napolitano and Montuori, 2025). Thus, we hypothesized that endothelial mitochondria were a source of inflammation-induced formyl peptide release, leading to increased neutrophil recruitment. To test this hypothesis, we designed an in vitro trans-well assay to quantify neutrophil transmigration. HLMVECs were treated with TNFα or vehicle control for 1 hr, washed to remove residual cytokine, and incubated in low serum media for an additional 24 hr. The resulting conditioned medium was collected, filtered, and placed into the lower chambers of trans-well plates. HL-60-derived neutrophils (referred to as dHL-60) were added to the upper chambers, and movement across the trans-well membrane was measured. Conditioned media from TNFα-treated endothelial cells induced significantly higher dHL-60 transmigration than conditioned media from control cells (Figure 5A). Moreover, conditioned media from TNFα-exposed endothelial cells also induced significantly higher Erk phosphorylation in dHL-60 cells (Figure 5B and C). Synthesized human mitochondrial formyl peptide (fMIT) also induced dHL-60 transmigration, indicating that FPR activation by mitochondria-derived formyl peptide can drive dHL-60 transmigration (Figure 5—figure supplement 1A). Additionally, fMIT induced Erk phosphorylation in dHL-60 cells to a similar extent as the bacterial formyl peptide fMLP (Figure 5—figure supplement 1B and C).

**Figure 5—figure supplement 1.**
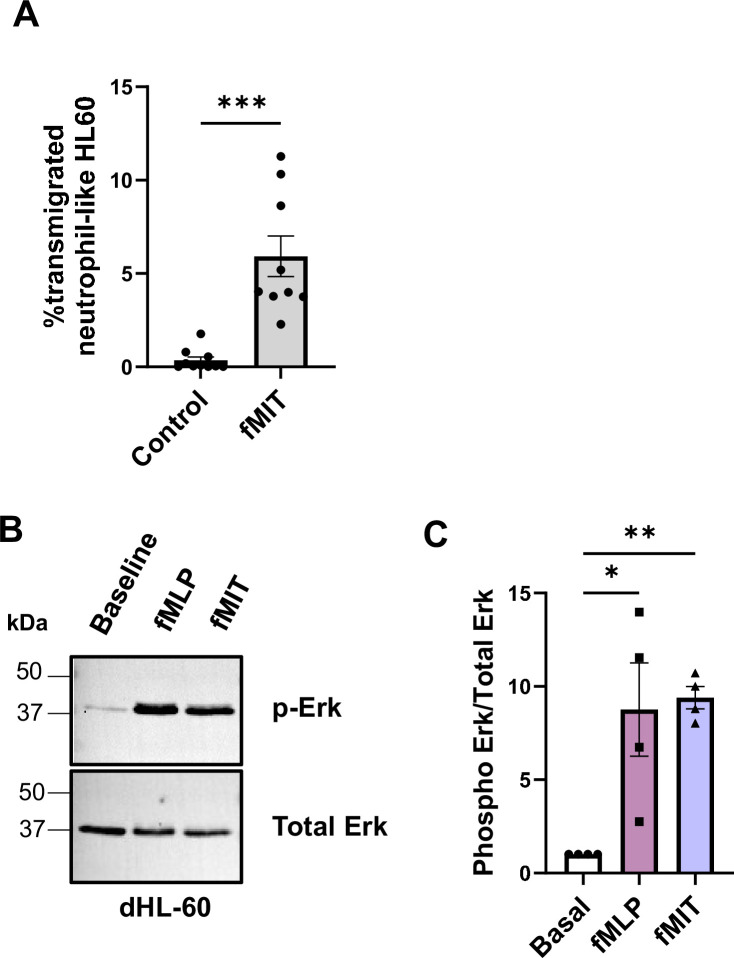
Mitochondrial formylated peptide induces trans-well migration of dHL-60 cells. Purified mitochondrial formyl peptide (fMIT, 100 nM) was added to the lower chamber of a trans-well assay plate. Differentiated HL-60 cells (dHL-60) were placed in the upper chamber. Transmigration in response to media from TNFα-treated and control cells was quantified (**A**). Statistical analysis was performed using Welch’s t-test. dHL-60 cells were exposed to 10 nM fMLP or fMIT for 10 min, lysed, and evaluated for Erk phosphorylation by western blot (**B, C**).Data in graphs are presented as mean ± SEM, and statistical significance was assessed by one-way ANOVA. Source data for (A) and (C) are available in Figure 5—figure supplement 1—source data 1. Uncropped western blot images for (B) are provided in Figure 5—figure supplement 1—source data 2. Figure 5—figure supplement 1—source data 1.Spreadsheet containing source data for Figure 5—figure supplement 1A and C. Figure 5—figure supplement 1—source data 2.Uncropped western blot images for Figure 5—figure supplement 1B, with relevant bands indicated. Figure 5—figure supplement 1—source data 3.Original western blot images for Figure 5—figure supplement 1B.

To determine whether formyl peptides were among the factors released by endothelial cells, we assessed the presence of formylated proteins in the cell culture medium. The mitochondrial protein ND6 is one of the thirteen proteins encoded in the mitochondrial DNA and is thus commonly used as an indicator for the presence of mitochondrial formylated proteins (Gabl et al., 2018; Kwon et al., 2021). We thus performed an ELISA to measure relative ND6 levels in the cell culture medium of HLMVECs treated with TNFα. TNFα induced an ~30% increase in ND6 levels released by endothelial cells (Figure 5D).

Given that cultured HLMVECs release ND6 in response to TNFα, we next investigated whether ND6 is similarly released into circulation in vivo during endotoxemia. Mice were injected intraperitoneally with a sub-lethal dose of LPS, and blood plasma was collected at 0, 3, 6, and 24 hr post-injection. Plasma proteins were precipitated using trichloroacetic acid (TCA), and the concentrated proteins were analyzed by Western blot. Interestingly, the observed molecular weight for murine ND6 was lower than previously reported for human plasma, possibly due to TCA precipitation or processing prior to release. LPS induced a significant increase in plasma ND6 levels at 6 hr post-injection, which was further increased at 24 hr (Figure 5E and F). Notably, this 6 hr time point coincides with endothelial mitophagy and early neutrophil recruitment to the lung.

Lastly, we examined whether the presence of ND6 in the blood was dependent on endothelial Pink1. We collected plasma samples from WT and *Pink1* EC-KD mice 6 hr after an intraperitoneal injection of a sub-lethal LPS dose. Compared to WT mice, *Pink1* EC-KD had significantly lower levels of LPS-induced plasma ND6 (Figure 5G and H).

## Discussion

**Figure 6.**
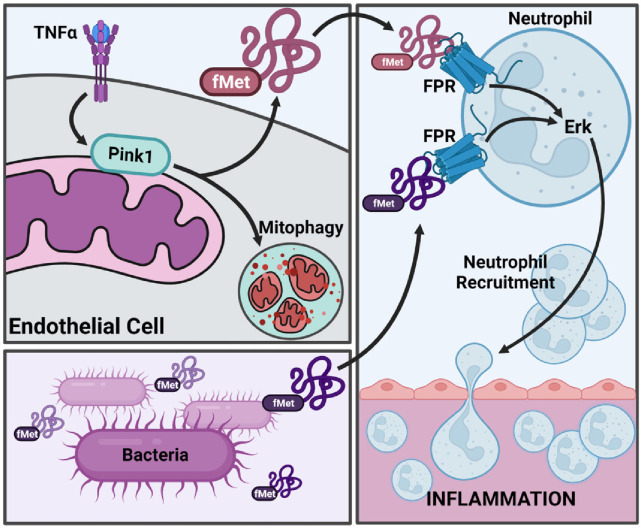
Inflammation-induced endothelial Pink1 activity releases formylated proteins to enhance inflammation. In response to inflammatory stimulus, Pink1 is activated in endothelial cells, leading to mitophagy and release of mitochondrial proteins such as ND6, which contain a formylated methionine (fMet) at the N-terminus. Bacteria, which share a prokaryotic ancestor with mitochondria, also produce and release N-formyl proteins. Both mitochondrial and bacterial N-formyl proteins activate neutrophils through formyl peptide receptors (FPRs), leading to increased Erk phosphorylation and increased neutrophil recruitment. Excessive neutrophil recruitment leads to increased aberrant inflammation. This schematic was created using BioRender.com.

In this study, we uncovered a novel pro-inflammatory role of Pink1 in endothelial cells. Inflammatory activation induced mitophagy in endothelial cells, both in vitro and in vivo using an endotoxin-induced lung injury model, which is characterized by excessive endothelial inflammation and influx of neutrophils (Bachmaier et al., 2007; Kolaczkowska and Kubes, 2013; Zhang et al., 2022). The observed endothelial mitophagy was mediated by Pink1 activation, and loss of Pink1 results in reduced mitophagy. Deletion of *Pink1* in endothelial cells resulted in increased survival in endotoxin-injected mice and significantly reduced neutrophil recruitment and activation in the lung. Lastly, we found that in response to inflammation, endothelial cells released ND6, a formylated mitochondrial protein and potent recruiter of neutrophils. Endothelial deletion of *Pink1* significantly reduced plasma ND6 levels, indicating not only that ND6 release is Pink1-dependent, but importantly, that endothelial cells are a significant source of circulating mitochondrial formyl peptide (Figure 6).

Lung endothelial cells displayed increased mitophagy in response to inflammatory mediators both in vivo and in vitro, as visualized using the mitophagy biosensor mt-Keima. Mt-Keima has been previously used to examine whole lung mitophagy (Sun et al., 2015), making it difficult to ascertain the fraction of endothelial mitophagy. Thus, to measure mitophagy specifically in the endothelium, we developed a method to isolate the endothelial mt-Keima signal from the rest of the lung in whole-organ imaging. Employing this method, we found that LPS significantly induced mitophagy in the mouse lung vascular endothelium. The inflammatory mediator TNFα, which is produced by immune cells in response to LPS, induced mitophagy in primary human lung endothelial cells.

Using an endothelial-specific Cas9 mouse model, and nanoparticle delivery of *Pink1* sgRNA, we were able to generate mice that lacked Pink1 expression specifically in endothelial cells, circumventing the confounding effects of global mitophagy knockouts. A surprising finding of this study was that deletion of *Pink1* in endothelial cells drastically reduced endotoxin-induced death in mice, indicating a pro-inflammatory role of endothelial Pink1. By contrast, mitophagy in other immune cells is typically associated with reduced inflammatory activation (Harris et al., 2018; Sliter et al., 2018), such as in macrophages where mitophagy is linked to reduced inflammasome activation and IL-1β production (Zhong et al., 2016). Thus, mitophagy appears to have uniquely pro-inflammatory consequences in inflamed endothelial cells. Interestingly, a subpopulation of hepatic endothelial cells characterized by a Pink1-driven, high-mitophagy signature was found to have increased activation of the pro-inflammatory macrophage migration inhibitory factor pathway (Pan et al., 2024). In our study, deletion of endothelial Pink1 significantly reduced LPS-induced neutrophil recruitment to the lung and subsequent IL-1β production. Endothelial mitophagy thus appears to be a mode of communication through which the endothelium regulates the immune response.

Recruitment of neutrophils by endothelial cells is partly controlled by expression of the adhesion molecule ICAM-1. However, we did not observe any significant impact of *Pink1* deletion on endothelial ICAM-1 expression. We thus sought an alternative explanation for how the deletion of a mitochondrial protein may alter neutrophil recruitment. We found that endothelial cells release mitochondrially encoded proteins, such as ND6, in response to inflammatory stimuli. These formylated proteins have been shown to be elevated in patient serum during sepsis, where higher circulating ND6 levels are correlated with higher mortality (Kwon et al., 2021). Mitochondrial formylated peptides were also found to be elevated in patients with COVID-19, leading to activation of neutrophils through FPR1 (Kuley et al., 2023). Similarly, mitochondrial formylated peptides were found to be increased in the serum of patients with ARDS (Lu et al., 2025). Here, we found that a significant portion of inflammation-induced ND6 is dependent on endothelial Pink1.

The link between mitophagy and release of mitochondrial DAMPs is typically considered to be an inverse one, as mitophagy generally reduces mitochondrial stress. However, several recent studies have pointed to mitochondrial stress triggering the release of mitochondrial fragments through autophagic machinery. For instance, mitochondria in acidic lysosomes, or mitoysosomes, are released by dopaminergic neurons and astrocytes through lysosome exocytosis following Flunarizine-induced Parkinsonism (Bao et al., 2022). Similarly, cardiomyocytes release mitochondria and mitochondrial fragments in an autophagy-dependent manner during cardiac stress (Nicolás-Ávila et al., 2020). Alternatively, mitochondria can also be expelled in extracellular vesicles (EVs) when lysosomal acidification is impaired (Liang et al., 2023). The mechanism of release of mitochondrial peptides, fragments, and organelles may thus be cell type and context dependent.

The inflammation-induced release of formylated peptides raises an intriguing question about the relationship between mitochondria, evolutionary endosymbionts, and their eukaryotic hosts. Despite the ancient origins of our dependence on mitochondria, why are mitochondria still recognized as foreign by the immune system? Formylation of mitochondrial proteins is required for their function, as deletion of formyl-transferase leads to decreased efficiency of oxidative phosphorylation (Tucker et al., 2011; Arguello et al., 2018). Perhaps inflammatory activation by mitochondrial formyl-peptides can be beneficial to activate the immune system in the setting of host defense when tissue injury is occurring and leading to the release of endogenous mitochondrial formyl peptides. The increased mortality correlated with higher ND6 levels in patients with sepsis (Kwon et al., 2021) suggests that in the setting of hyperinflammation such as lung injury, the pro-inflammatory detrimental effects may override other potential host defense benefits such as amplified activation of inflammatory pathways. Mammalian immune systems may have evolved neutrophil sensing of mitochondrial formylated peptides released by the endothelium as a means of activating neutrophils even before neutrophils come into direct contact with bacteria and their formylated proteins. Such priming of neutrophils when they transmigrate across the endothelial barrier by mitochondrial formylated peptides could be an essential determinant of subsequent bacterial elimination by neutrophils. However, our work suggests that there may be a need to prevent such priming when the excessive inflammation and activation of neutrophils such as in the case of inflammatory lung injury becomes an even bigger liability than the benefits of amplified neutrophil activity.

## Materials and methods

### Materials

Human recombinant TNFα was obtained from R&D Systems (Cat# 210-TA-020). Antibodies against Pink1 (Cat# 6946 S), p44/42 MAPK (Total Erk; Cat# 4695 S), and phospho-p44/42 MAPK (p-Erk; Thr202/Tyr204; Cat# 4370 S), and LC3b (Cat# 2775 S) were obtained from Cell Signaling Technologies. ND6 antibody (Cat# NBP2-94464) was obtained from Novus Biologics. CD31 antibody (Cat#553370) was obtained from BD Pharmingen. β-Actin (Cat# sc-47778 HRP), Tomm20 (Cat# sc-17764), and VE-Cadherin (Cat# sc-9989) antibodies were from SantaCruz Biotechnology. IL-1β antibody (Cat# MM425B) was obtained from Thermo Fisher Scientific. Lamp1 (Cat# ab25245) was obtained from Abcam. The secondary anti-mouse (Cat# 115-035-174) and anti-rabbit (Cat# 111-036-003) antibodies were obtained from Jackson&ImmunoResearch. LPS and FCCP were obtained from Millipore Sigma. Griffonia Simplicifolia Lectin I (GSL I) Isolectin-B4, DyLight 649 was obtained from Vector Labs (Cat# DL-1208). AlexaFluor 647 anti-rabbit (Cat# A32795) and AlexaFluor 488 anti-mouse (Cat# A21202) were obtained from Invitrogen. Fluorescent conjugated antibodies for flow cytometry were obtained from Biolegend - BV421-CD31 (Cat# 102423), PE-CD54 (ICAM-1, Cat# 116108), APC-CD45 (Cat# 103112), BV421-Ly6G (Cat# 127627), PE-CD11b (Cat# 101207), Bv421 Isotype Control (Cat# 400259), APC Isotype Control (Cat# 400612), and PE Isotype Control (Cat# 400608). Purified fMLP (Formyl-Met-Leu-Phe, Cat# ab141806) was obtained from Abcam and fMIT (Formyl-Met-Met-Tyr-Ala-Leu-Phe, Cat# 005–48) was obtained from Phoenix Pharmaceuticals.

### Cell culture

Primary HLMVECs (Cell Applications Cat# 540–05 a) were cultured in flasks coated with 0.2% gelatin, using Endothelial Basal Medium 2 (Lonza Cat# CC-3156) supplemented with 10% FBS (Hyclone) and Microvascular Endothelial Growth Medium growth factor kits (Lonza Cat# CC-4147). HLMVECs between passages 5 and 9 were used for experiments. HEK293T cells (ATCC, Cat# CRL-3216) were cultured in DMEM (Corning) supplemented with 10% FBS and 1% Pen-Strep (Corning). HL-60 cells were cultured in RPMI media (Corning) containing glutamine and supplemented with 10% FBS, 1% Pen-Strep, and 20 mM HEPES. HL-60 cells (ATCC, Cat# CCL-240) were differentiated to neutrophil-like dHL-60 cells by supplementing media with 1.3% DMSO for 5–6 days. HEK293T and HL-60 were purchased directly from ATCC, which were authenticated using short tandem repeat (STR) profiling. Both cell lines and primary HLMVECs were confirmed to be negative for mycoplasma contamination prior to distribution. We use low passage numbers (<15 passages for cell lines) and routinely monitor under microscopy to ensure expected morphology.

### Virus generation

Lentivirus for mt-Keima was generated by co-transfecting the lentiviral plasmids in HEK293T cells with VSV-G (the envelope expressing plasmid, Addgene, #12259), psPax2 (the virus packaging plasmid, Addgene, #12260) using JetPrime transfection reagent (Polyplus) as per the manufacturer’s protocol. Cell culture supernatant was collected 48 and 72 hr after transfection, and viral particles were precipitated using Lenti-X concentrator (Takara Bio) following the manufacturer’s protocol. HLMVECs were transduced with lentivirus in media containing 4 µg/mL Polybrene (Santa Cruz Biotechnology), and expression was observed 2–4 days following infection.

### Immunofluorescence and confocal microscopy

HLMVECs expressing mt-Keima were plated on gelatin-coated glass-bottom dishes (Matek) 24 hr prior to visualization. Cells were treated as indicated and imaged live using a Zeiss Laser Scanning 710 BiG microscope equipped with a Plan-Apochromat 63 x/1.40 NA Oil DIC objective (Zeiss) and GaAsP detectors, at 37 °C with 5% CO_2_. Mitophagy was calculated as the percentage of mitolysosome area compared to total mitochondria (mitolysosomes + cytoplasmic mitochondria).

TMRM (Invitrogen, Cat# T668) was used to measure the mitochondrial membrane potential. First, HLMVECs were plated on gelatin-coated glass-bottom dishes and treated with TNFα (10 ng/mL) for the indicated times. 30 min prior to imaging, cells were loaded with 20 nM TMRM, then washed once with HBSS with Calcium and Magnesium before imaging on a confocal microscope.

To measure mitochondrial Pink1, HLMVECs were plated on gelatin-coated glass-bottom dishes and treated as indicated. Cells were then washed, fixed in 4% paraformaldehyde (PFA), and permeabilized using 0.01% Triton X-100. After blocking in PBS buffer with 10% Donkey serum plus 2% BSA, cells were then stained using primary antibodies against Pink1 (Cell Signaling Technologies, Cat# 6946 S) and Tomm20 (Santa Cruz, Cat# sc-17764), with AlexaFluor 647 anti-rabbit (Invitrogen, Cat# A32795) and AlexaFluor 488 anti-mouse (Invitrogen, Cat# A21202) used as secondary antibodies. DAPI was used to stain the nucleus. Cells were then visualized on a confocal mode of the Zeiss LSM900 microscope to visualize mitochondria and Pink1. Two-dimensional reconstructions of the z-stacks were prepared using Imaris image analysis software. For quantification of mitochondrial Pink1, confocal images were taken on a Zeiss LSM900 microscope. Following background corrections, the Mander’s coefficient of Tomm20 overlapping with Pink1 was calculated per image using the JACOP plug-in for ImageJ, representing the proportion of Pink1 that is mitochondrially localized. To compare the total amount of Pink1 in the mitochondrial compartment, a mask of the mitochondrial network was created based on the Tomm20 channel. The intensity of Pink1 signal within the mitochondrial mask was normalized to the Tomm20 signal to account for variation in mitochondrial density. Average Pink1 intensity was multiplied by the area of Pink1 to give the total mitochondrial Pink1 per visual field. Graphed data points represent the average Mander’s coefficient or total mitochondrial Pink1 intensity over 6–10 visual fields within a single replicate experiment.

For ex vivo imaging, mt-Keima mice (obtained from Dr. Toren Finkel’s lab) were injected with LPS (8 mg/kg, i.p.) 6 hr prior to analysis. 30 min prior to lung collection, anesthetized mt-Keima mice aged 8 weeks were injected with 50 µg of Isolectin-B4 (in 100 µL of PBS) retro-orbitally to stain the mouse endothelium. At the indicated time, the mouse lung was perfused with PBS. Lungs were transferred to glass-bottomed dishes in HBSS and immediately imaged whole by confocal microscope. All images were taken within 1 hr of harvest. Images were quantified by generating a mask based on Isolectin-B4 staining for the endothelium. The mask was applied to a ratiometric mask of cytoplasmic (Excitation: 488 nm) to lysosomal (Excitation 560 nm) mt-Keima. The image was thresholded and the area of mitophagy was quantified. 10–20 fields of view were quantified per lung, four mouse lungs per group, from four independent experiments. Data represents the area of mitolysosomes, defined as regions where the ratio of neutral: acidic mt-Keima was above the threshold. %Mitophagy was calculated by normalizing mitolysosome area for LPS-injected mice to that of the corresponding PBS-injected mice within each experimental replicate.

For mitophagy staining in mouse lung sections, Cas9-VECre mice were intranasally administered with zwitterionic EV complexes with 75 nmoles of either *Pink1* sgRNAs for EC-specific *Pink1* knockdown, or control sgRNA. 4 days later, mice were intraperitoneally injected with LPS at a dose of 8 mg/kg body weight. 6 hr following treatment, lungs were perfused and harvested. The right four lobes were used for endothelial cell isolation and subsequent analysis. The left lung was fixed in 4% PFA overnight and used for immunostaining. Lungs were cryoprotected in 30% sucrose for an additional 2 days before embedding in OCT (Optimal Cutting Temperature). The slides were cryo-sectioned in 10 μm sections. The sections were permeabilized using 0.2% Triton X-100 for 10 min and blocked in blocking buffer (PBS +10% goat serum + 2% BSA+0.05% Tween 20) for 1 hr. The slides were then stained with antibodies against Lamp1 (Abcam, Cat# ab25245) and Tomm20 (Santa Cruz Biotechnology, Cat# sc-17764). Nuclei were stained using DAPI. Slides were imaged on a confocal microscope (Zeiss LSM700). The area of colocalization of Tomm20 and Lamp1, indicating lysosomal mitochondria, was quantified as a fraction of total mitochondria. Each data point represents the average fraction of lysosomal mitochondria measured in three to six areas of a single mouse lung. Results comprise n=3 independent experiments.

### Western blotting

Treated HLMVECs, isolated endothelial cells, or non-endothelial cells were lysed in cell lysis buffer (50 mM HEPES pH 7.5, 120 mM NaCl, 5 mM EDTA, 10 mM Na pyrophosphate, 50 mM NaF, 1 mM Na_3_VO_4_, 1% Triton X-100) supplemented with protease inhibitor cocktails (ThermoFisher Scientific, Cat# 78430) and phosphatase inhibitor cocktails (Millipore Sigma, Cat# 524625). For mouse lung tissue samples, the post-caval lobe was flash frozen in dry ice immediately after harvesting. On thawing, tissue was homogenized (NextAdvance Bullet Blender) in lysis buffer to extract protein. Western blotting was performed as previously described, using 1:1000 dilution for all antibodies except β-actin (1:5000). Western blots were imaged using an iBright CL1500 machine (ThermoFisher). For total protein staining, blots were washed with water three times, incubated in Ponceau S (Sigma, Cat# P7170) for 5 min. Blots were washed with water three times before imaging.

### fMLP and fMIT treatment of dHL-60 cells

After differentiation in 1.3% DMSO, dHL-60 cells were serum-starved in RPMI media containing glutamine, supplemented with 0.1% FBS (RPMI-SFM) for 2 hr. 5 × 10^5^ cells were then treated with either DMSO, 10 nM fMLP, or 10 nM fMIT for 10 min. Cells were then spun down and lysed for analysis of Erk and phospho-Erk by western blot.

### Treating dHL-60 cells with endothelial conditioned media

HLMVECs were plated on gelatin-coated plates and allowed to grow to confluency overnight. Cells were then serum-starved in EBM2 media supplemented with 0.1% FBS and treated with 10 ng/mL TNFα or vehicle control for 1 hr and washed and replaced with EBM2 media supplemented with 0.1% FBS and incubated for an additional 23 hr. Conditioned media was then collected and passed through 0.44-μm syringe filter to remove cell debris. Differentiated HL-60 cells were serum-starved in RPMI-SFM for 2 hr, then spun down and resuspended in endothelial conditioned media for 10 min. Lysate was collected and used for analysis of Erk and phospho-Erk by western blot.

### Neutrophil transmigration assay

dHL-60 cells were suspended in RPMI Migration Buffer (RPMI 1640 + 2 mM L-Glutamine, 20 mM HEPES +0.1% BSA Fraction V) at a density of 2 × 10^6^/ mL. 100 μL of dHL-60 were added to the upper chamber of the trans-well insert in a 96-well trans-well plate (Corning, Cat# 3387). 100 nM fMIT, DMSO vehicle, or conditioned media was added to the bottom chamber. Following a 1 hr incubation, both the migrated dHL-60 cells in the bottom chamber and the input dHL-60 cells were counted using a Beckman CytoFlex S Flow Cytometer. The % transmigrated dHL-60 was calculated by normalizing the transmigrated dHL-60 to the total input dHL-60.

### Detecting ND6 in endothelial culture medium

HLMVECs were treated with 10 ng/mL TNFα for 24 hr. Cell culture medium was collected and filtered through 0.45 µm filters to remove cell debris, and flash frozen in dry ice. ND6 was detected in samples using an ELISA kit (MyBiosource, MBS936598) as per manufacturer’s protocol, using undiluted media samples. Absorbance at 450 nm was measured using a FlexStation III plate reader (Molecular Devices). Samples were normalized to untreated controls.

### Animal procedures

All animal experiments were conducted in accordance with NIH guidelines for the care and use of Laboratory Animals and were approved by the IACUC of the University of Illinois. C57BL/6 mice (strain# 000664) were purchased from Jackson Lab. Cas9-VEcre mice were generated by crossing knock-in Cas9 mice (Jackson Lab, Strain# 026175) and VE-cadherin-Cre (Ve-Cre) mice that constitutively express Cre under the endothelial-specific promoter CDH5 (Jackson lab, Strain# 006137). Mt-Keima mice were obtained from Dr. Toren Finkel’s lab. All experiments were performed using 8- to 10-week-old animals using age-matched and sex-matched groups. Mice were intraperitoneally administered with LPS at a lethal dose (10 mg/kg) for survival and sub-lethal dose (8 mg/kg) for all other experiments. For retro-orbital injections, mice were anesthetized with 2% isoflurane inhalation at flow rate of 0.6 l/min. For endpoint experiments, mice were anesthetized with intraperitoneal administration of a mixture of Ketamine (100 mg/kg), Xylazine (2 mg/kg), and acepromazine (2 mg/kg) in saline solution before blood collection and lung harvesting.

### Detecting ND6 in mouse plasma

Blood samples from control and Pink1^-/-^ mice were collected via heart puncture in heparin-coated syringes. The blood samples were centrifuged at 1500 × *g* for 15 min and the supernatants were collected for plasma. For ND6 evaluation, plasma proteins were concentrated using 13% TCA, and pelleted protein was resuspended in 4 x Laemmli Sample Buffer (Bio-Rad Laboratories) supplemented with Tris pH 8.0 (Invitrogen) to neutralize any residual TCA. Samples were analyzed by western blot for the presence of ND6 (Novus Biologics, Cat# NBP2-94464). Band intensity of ND6 was quantified using Fiji software and normalized to PBS-injected control mice. Ponceau S stains were used to confirm equal protein loading.

### Mouse endothelial cell isolation

Perfused mouse lungs were minced and digested in Type 1 collagenase (1 mg/mL in HBSS buffer with calcium and magnesium) for 45 min at 37 °C incubator with gentle shaking. Mixtures were titrated with #18 G needles, and the dissociated cells were then filtered through 40 μm disposable cell strainer to remove undigested clumps. RBC were lysed using RBC lysis buffer (eBioscience) for 2 min at room temperature. The isolated cells were incubated with 2.5 µg anti-CD31 antibody (BD, Cat#553370) in 0.5 mL suspension buffer (PBS +0.5% BSA+2 mM EDTA +4.5 mg/mL D-glucose) at 4  °C for 30 min with gentle tilting and rotation. After washing, cells were then incubated with pre-washed Dynabeads (Invitrogen, Cat #11035, 25 µL beads in 0.5 mL suspension buffer) at 4 °C for 25 min with gentle tilting and rotation. ECs were harvested by magnetic separation.

### Nanoparticle preparation and in vivo delivery to generate mice with endothelial-specific Pink1 depletion

Cas9-Vecre mice injected with nanoparticles containing *Pink1* sgRNAs were used to generate EC-specific Pink1 knockout mice.

### Liposome preparation for intravenous delivery of plasmid DNA

Age-matched C57BL/6 mice (Jackson, 000664) injected with Pink1 sgRNAs were used as controls. *Pink1* sgRNAs were designed and cloned to pGS plasmid (Genscript, #1 GCTGGTCCCGGCAAGCCGCG, #2 CAAGCGCGTGTCTGACCCAC). Liposomes were freshly made using DDAB and cholesterol as described (Orrington-Myers et al., 2006; Zhang et al., 2017). Briefly, a mixture of DDAB and cholesterol was dissolved in chloroform, a lipid layer was formed in an evaporator (Model R-124, Rotavapor), and 5% glucose solution was added to the flask to dissolve the lipid form. Multilamellar liposomes were formed via sonication for 60 min and passed through a 0.22 µm filter. 45 µg pGS-*Pink1* sgRNAs were gently mixed with liposome. A total volume of 150 µL of the mixture was injected in either Cas9-VECre mice or C57BL/6 mice via retro-orbital injection. 4 days later, mice were treated with LPS at 8 mg/kg body weight via intraperitoneal injection, using PBS as a vehicle control. Tissues were harvested for experiments at the indicated times. Depletion of *Pink1* was confirmed by western blot.

### Zwitterionic vesicle (ZV) preparation for intranasal delivery of sgRNA

ZVs were prepared using a lipid film hydration method. The lipids 1,2-dioleoyl-sn-glycero-3-phosphocholine (DOPC), 1,2-dioleoyl-sn-glycero-3-phosphoethanolamine (DOPE), and 1-palmitoyl-2-oleoyl-glycero-3-phosphocholine (POPC) were purchased in powder form from Avanti Polar Lipids. The zwitterionic ionizable lipid, Cholesteryl-arginine, was synthesized in-house. Briefly, cholesteryl chloroformate (Sigma, Cat# C77007) was reacted with L-arginine (Sigma, Cat# A8094) in chloroform using a catalytic amount of the organic base N,N-diisopropylethylamine (Sigma, Cat# 387649). The reaction mixture was stirred overnight, followed by column-based purification and lyophilization to obtain pure cholesteryl-arginine (>98%). All lipid powders were dissolved in tert-butanol at a final concentration of 2 mg/mL. Lipid solutions were mixed with 0.75 nmoles of *Pink1* (IDT, mA*mC*mC*rCrArCrUrGrGrArCrArCrUrCrGrArUrGrCrGrUrUrUrUrArGrArGrCrUrArGrArArArUr5ArGrCrArArGrUrUrArArArArUrArArGrGrCrUrArGrUrCrCrGrUrUrArUrCrArArCrUrUrGrArArArArArGrUrGrGrCrArCrCrGrArGrUrCrGrGrUrGrCmU*mU*mU*rU) or control sgRNA (IDT, Cat# 1072555) in excess tert-butanol and dried overnight under vacuum using a lyophilizer to form a thin lipid film. The final molar ratio of lipids was DOPC:DOPE:POPC:Cholesteryl-arginine=20:20:20:40, combined with 10 nmoles of sgRNA. The dried film was hydrated with 1 mL of PBS prepared in ribonuclease-free water for 15 min. The resulting liposomes were extruded through a polycarbonate membrane (0.1 µm pore size, Avanti Polar Lipids, Cat# 610005). 75 μL of ZV complexes were administered to each Cas9/VECre mouse intranasally, 4 days prior to use in further experiments. Depletion of *Pink1* was confirmed by western blot.

### Evans Blue Albumin (EBA) assay and wet/dry ratio to measure lung endothelial permeability and edema

45 min prior to lung collection, anesthetized mice aged 8 weeks were retro-orbitally injected with EBA (200 mg/kg body weight, 100 µL of 40 mg/mL EBA to 20 g mice). At the indicated times, the lungs were perfused with 10 mL of PBS at 5 mL/min. The whole lungs were removed and weighed. The whole lungs in PBS were ground and an equal volume of formamide was added to extract the EBA at 60 °C overnight. The mixture was centrifuged at 5000 × *g* for 30 min, and the absorbance of supernatants at OD620 and OD740 were measured. OD740 was used to exclude residual blood contamination, and the corrected A620 was calculated using the equation A620(corrected)=A620-1.426*A740+0.03. A calibration curve was generated using serial dilution of EBA and was used to calculate the amount of EBA leaked into the lung normalized to mouse body weight.

For wet/dry ratio, the lungs were harvested without perfusion, and the weight was measured. The tubes containing the lungs were dried in a 60 °C oven for 3 days and weighed. The ratio of wet/dry lung weight was then calculated.

### Flow cytometry analysis

For each sample, 2 × 10^5^ Isolated lung cells in 100 µL of cell stain buffer (Biolegend Cat#420201) were blocked using TruStain FcX (anti-mouse CD16/32) antibody (Biolegend, Cat#101319) for 10 min at 4 °C. Antibodies were added at a dilution of 1:100 in the combinations described, and cells were incubated at 4 °C for 30 min with gentle shaking. Cells were fixed by adding an equal volume of Fixation buffer (Biolegend Cat# 420801) for 10 min, washed, and then analyzed by flow cytometry. For endothelial ICAM-1 expression, the median fluorescence intensity of ICAM-1 was measured in CD45^-^CD31^+^ cells. To quantify neutrophil infiltration in the lung, the percent of CD45^+^Ly6G^+^ cells was measured by flow cytometry. The total number of cells in the lung was calculated manually using a hemocytometer to convert the percentage of neutrophils to the total number of neutrophils in the lung. Median fluorescent intensity of CD11b in CD45^+^Ly6G^+^ cells was measured to indicate neutrophil activation.

### Statistical analysis

Western blot band intensity and confocal microscopy images were quantified using ImageJ. Brightness and contrast of confocal microscopy images were adjusted for representative purposes only. Data is presented as mean ± SEM with significance levels expressed as ∗p<0.05, ∗∗p<0.01, ∗∗∗p<0.001, and ∗∗∗∗p<0.0001. All statistical analyses were performed using GraphPad Prism 11, by one-way ANOVA, with Holm-Sidak corrections for multiple comparisons, except where mentioned.

## Data Availability

Data are included in the manuscript and the supporting files. No new sequencing datasets were generated for this manuscript.

## Additional files

MDAR checklist
