# Supplementary figures and images for: Pink1-mediated mitophagy in the endothelium releases proteins encoded by mitochondrial DNA and activates neutrophil responses during inflammation
Source: eLife. 2026 Jul 1;15:e82205. doi: 10.7554/eLife.82205 (PMC13423353; doi:10.7554/eLife.82205)

Figure 2, figure supplement 2 A

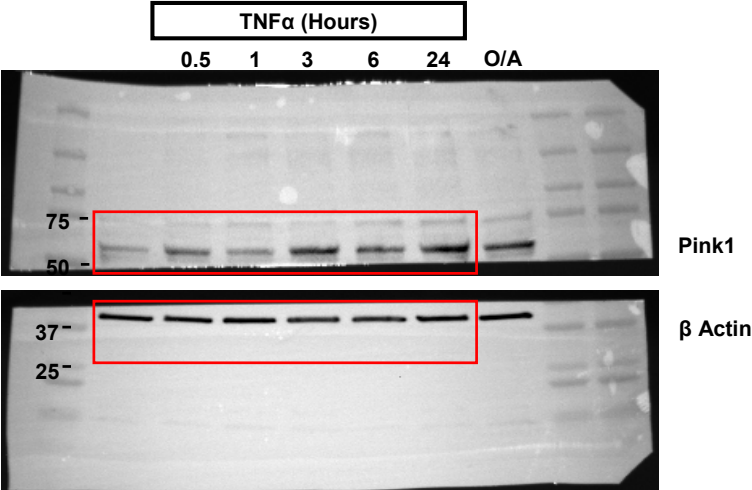

O/A: Oligomycin + Antimycin A

Supplement: Figure 2—figure supplement 2—source data 2. [file elife-82205-fig2-figsupp2-data2.pdf]

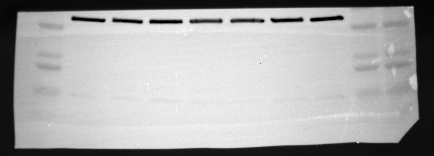

Supplement: Figure 2—figure supplement 2—source data 3. [file elife-82205-fig2-figsupp2-data3.zip › Figure 2 figure supplement 2 source data 3/Figure_2_Figure_Supplement_2A_Beta_Actin.tif]

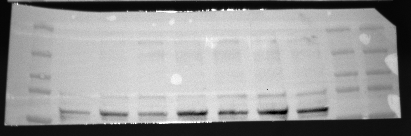

Supplement: Figure 2—figure supplement 2—source data 3. [file elife-82205-fig2-figsupp2-data3.zip › Figure 2 figure supplement 2 source data 3/Figure_2_Figure_Supplement_2A_Pink1.tif]

Figure 3 A:

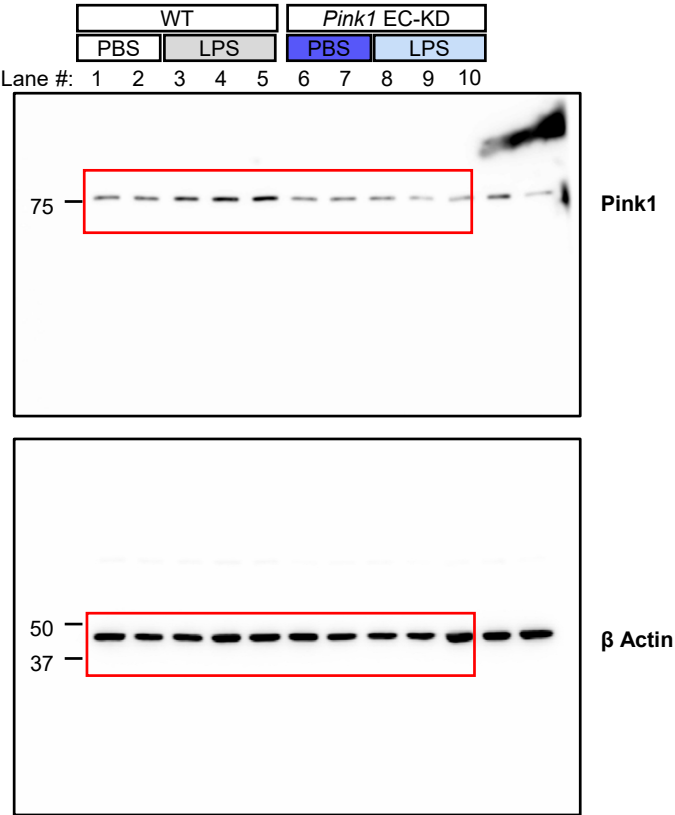

Figure 3 E:

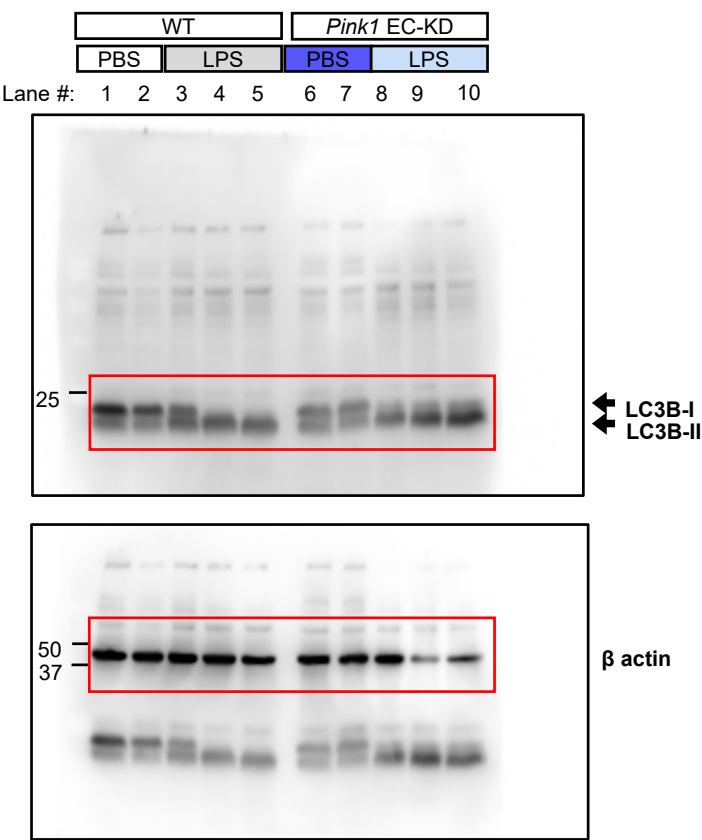

Supplement: Figure 3—source data 2. [file elife-82205-fig3-data2.pdf]

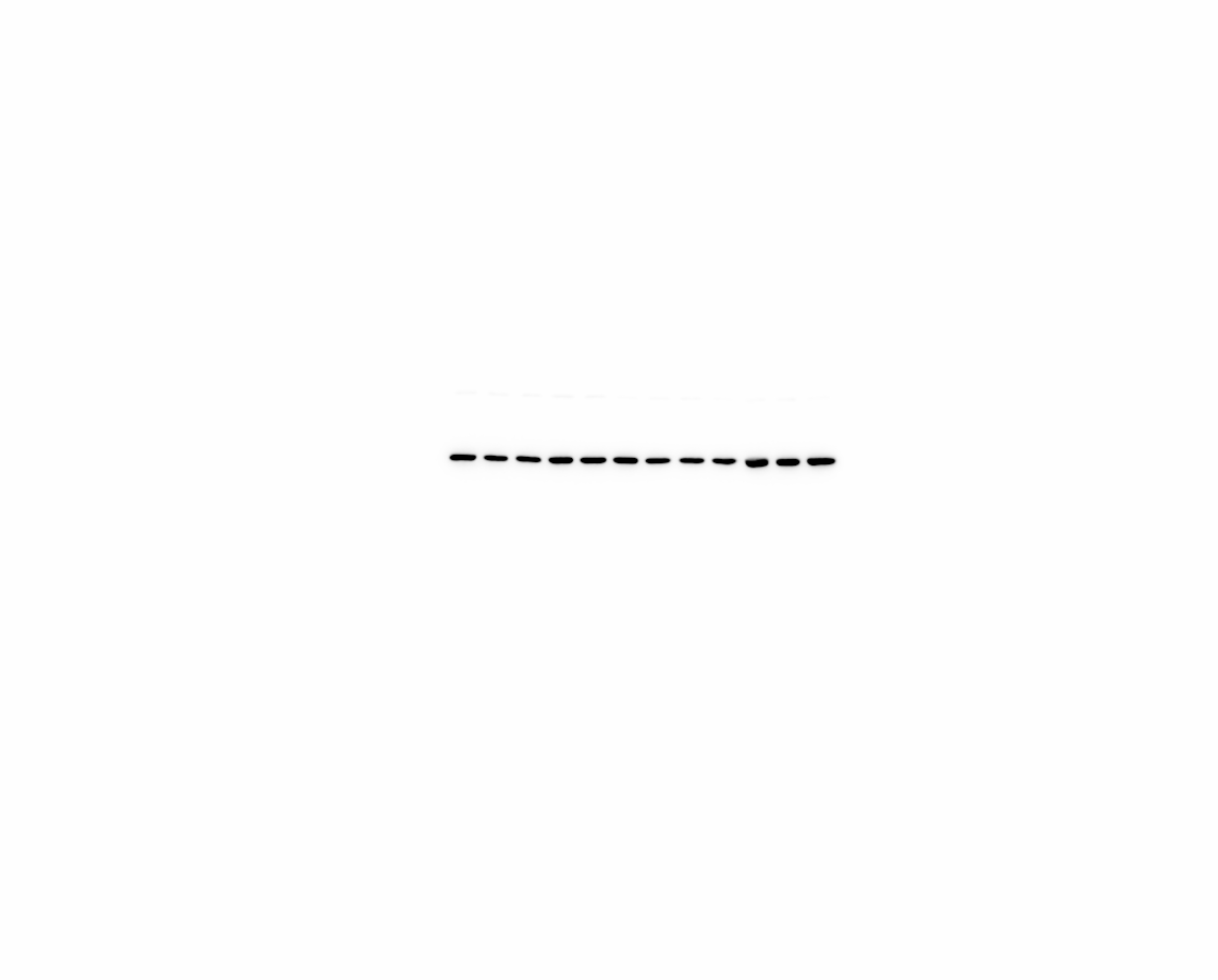

Supplement: Figure 3—source data 3. [file elife-82205-fig3-data3.zip › Figure 3 Source Data 3/Figure_3A_Beta_Actin.jpg]

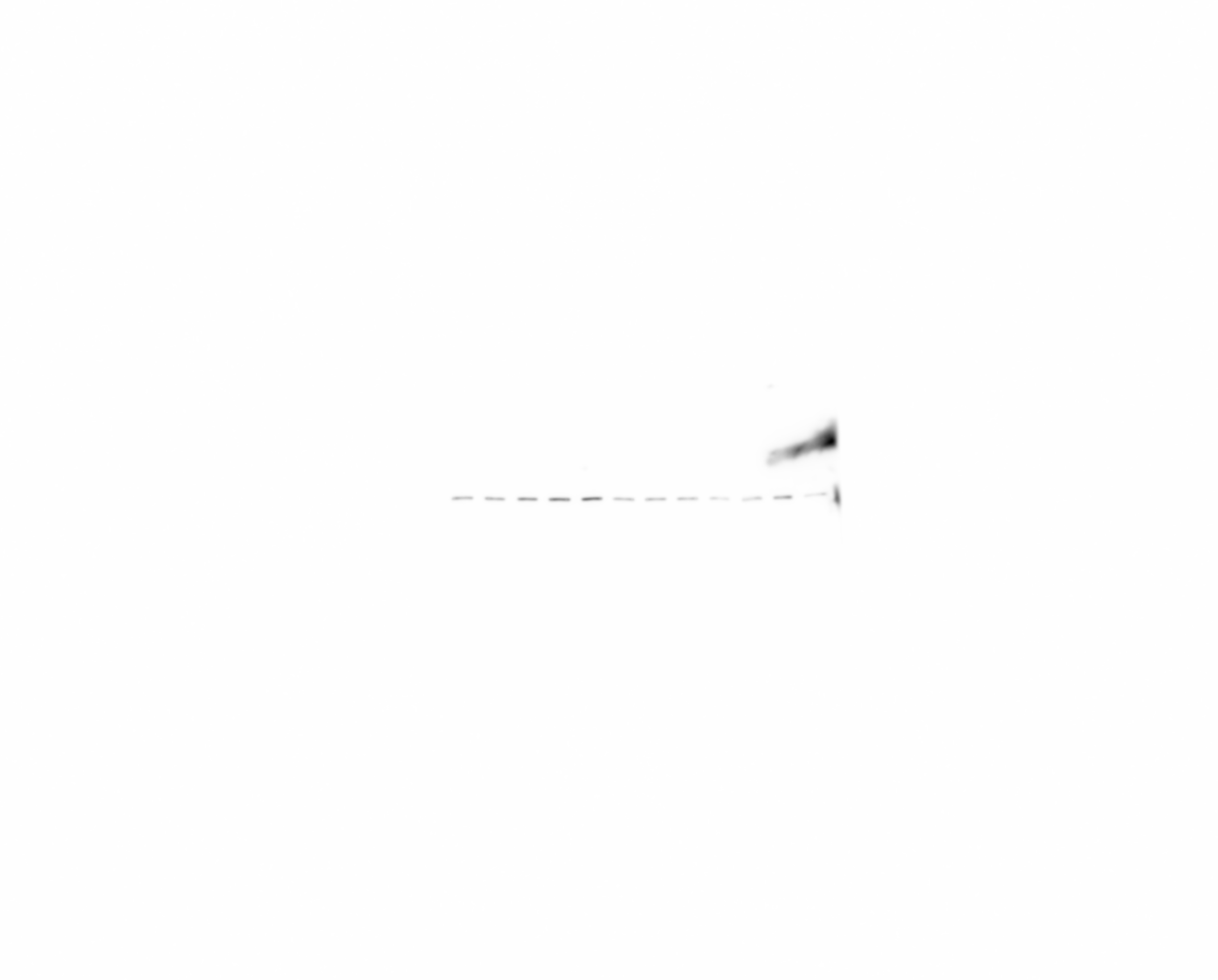

Supplement: Figure 3—source data 3. [file elife-82205-fig3-data3.zip › Figure 3 Source Data 3/Figure_3A_Pink1.jpg]

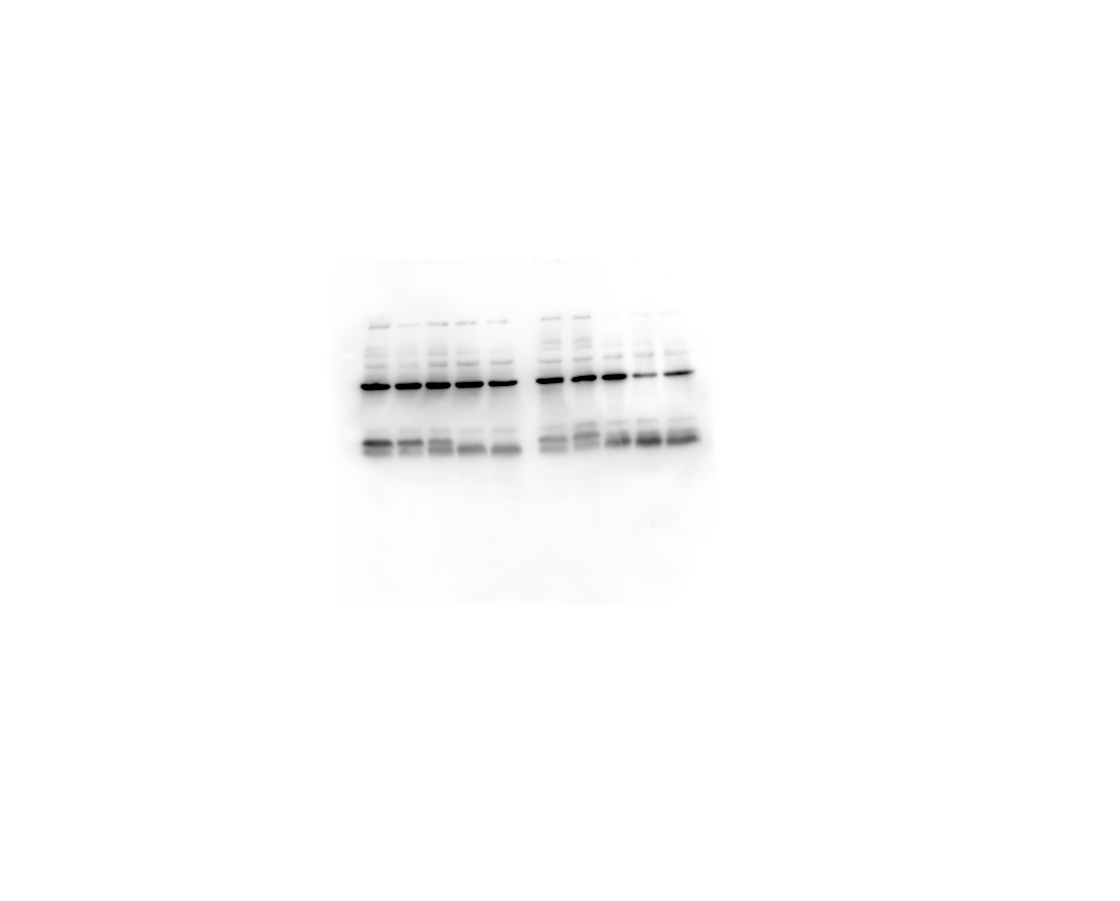

Supplement: Figure 3—source data 3. [file elife-82205-fig3-data3.zip › Figure 3 Source Data 3/Figure_3E_Beta_Actin.tif]

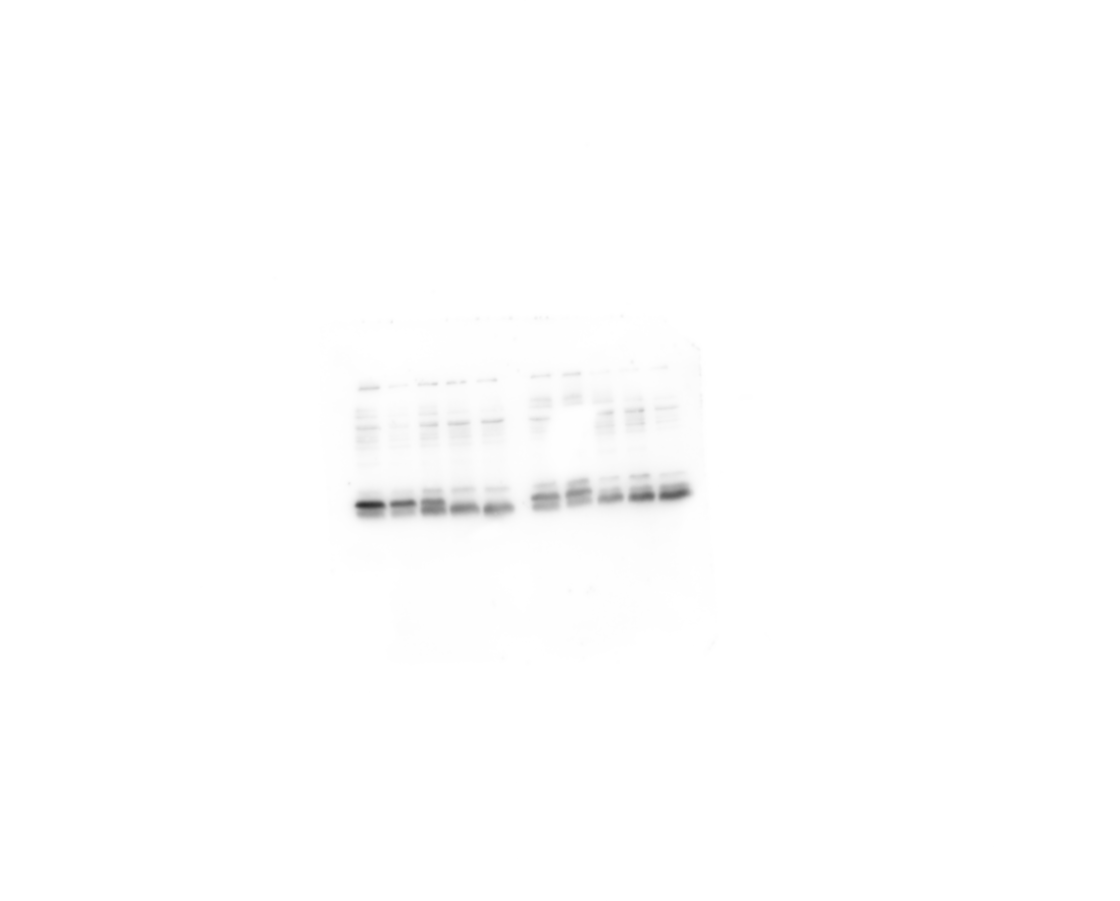

Supplement: Figure 3—source data 3. [file elife-82205-fig3-data3.zip › Figure 3 Source Data 3/Figure_3E_LC3b.tif]

Figure 3, figure supplement 2 A

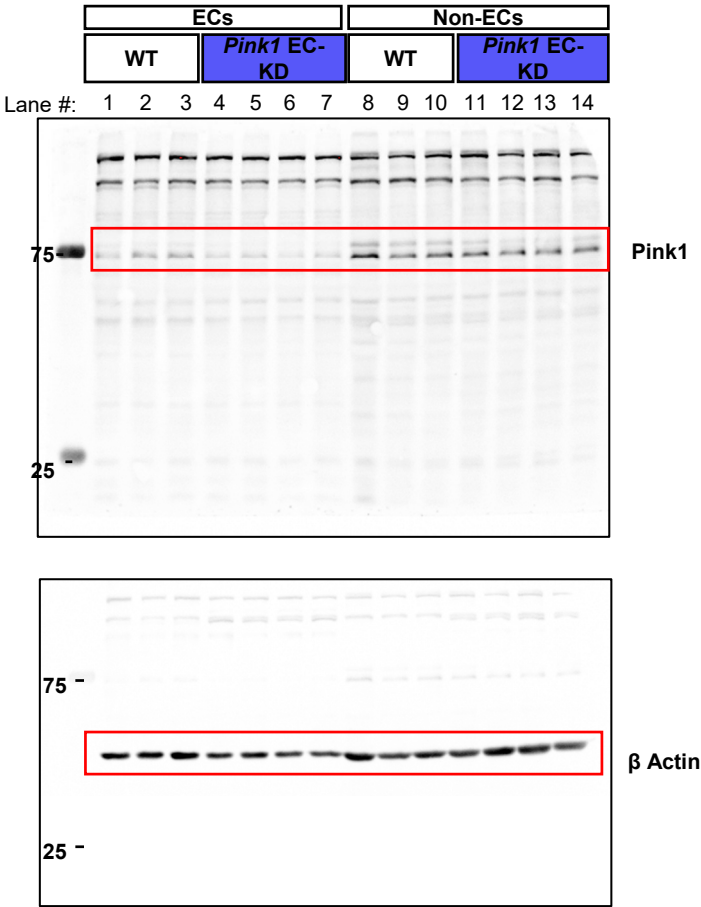

Supplement: Figure 3—figure supplement 2—source data 2. [file elife-82205-fig3-figsupp2-data2.pdf]

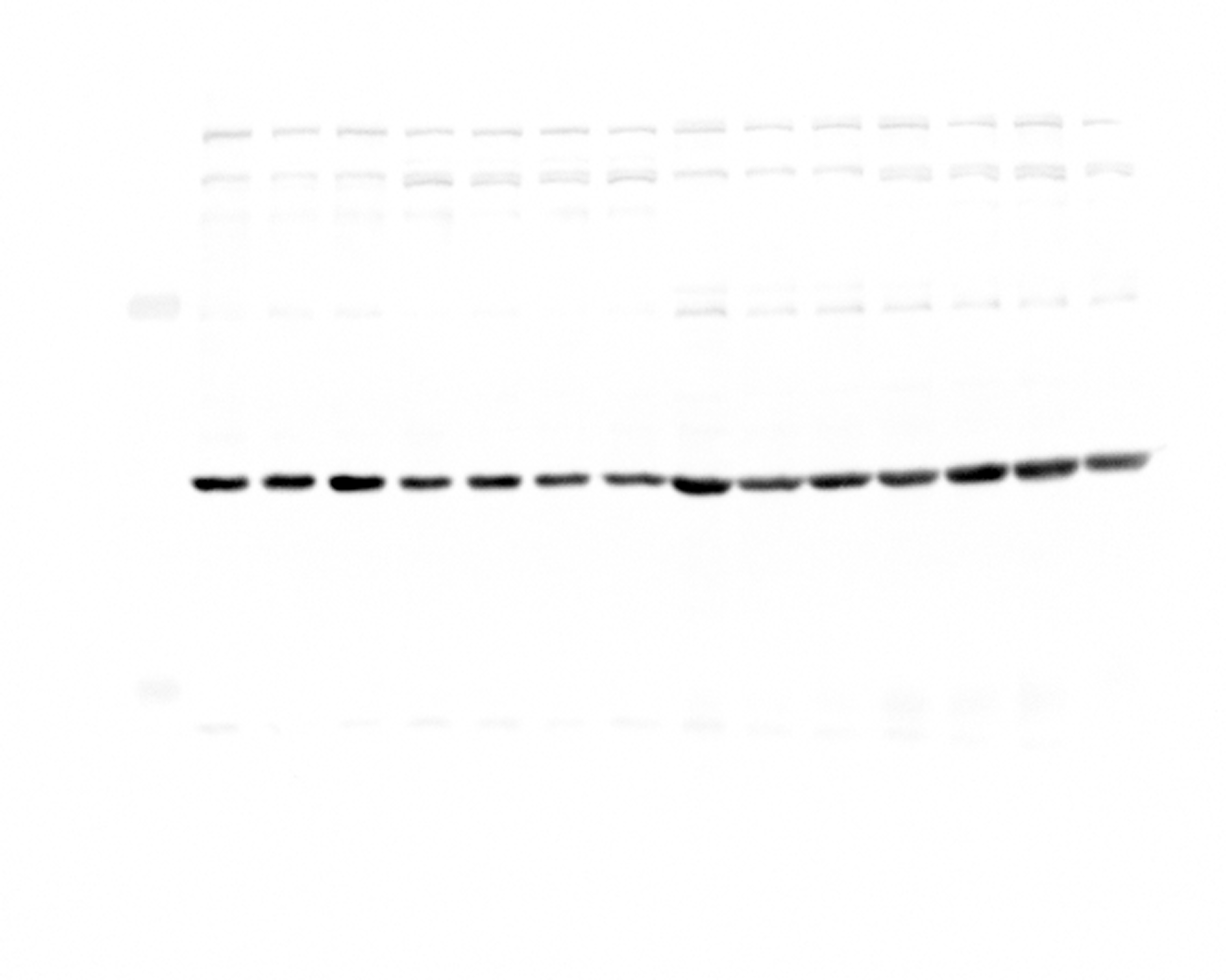

Supplement: Figure 3—figure supplement 2—source data 3. [file elife-82205-fig3-figsupp2-data3.zip › Figure 3 figure supplement 2 source data 3/Figure_3_Supplement_2A_Beta_Actin.tif]

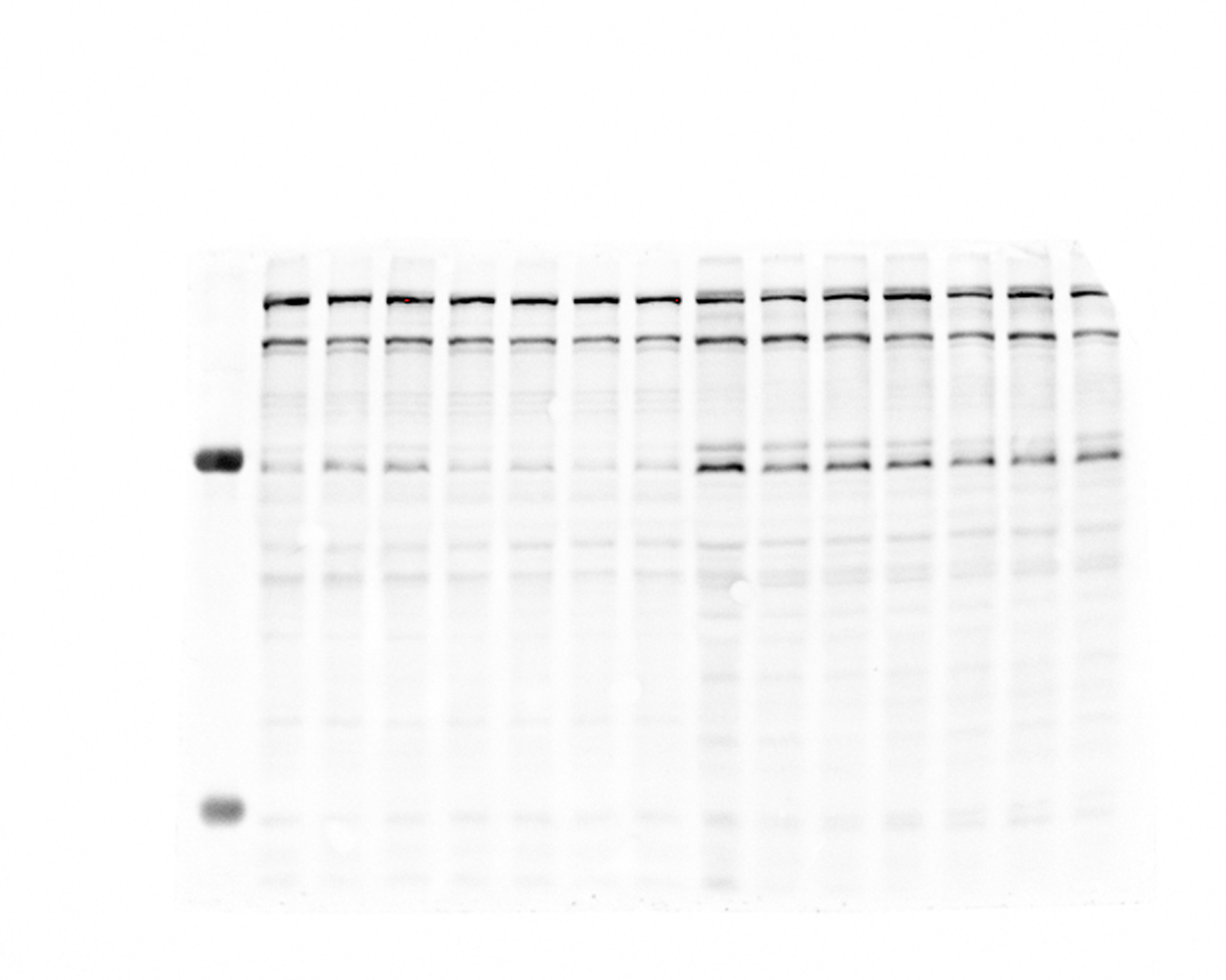

Supplement: Figure 3—figure supplement 2—source data 3. [file elife-82205-fig3-figsupp2-data3.zip › Figure 3 figure supplement 2 source data 3/Figure_3_Supplement_2A_Pink1.tif]

Figure 4 F:

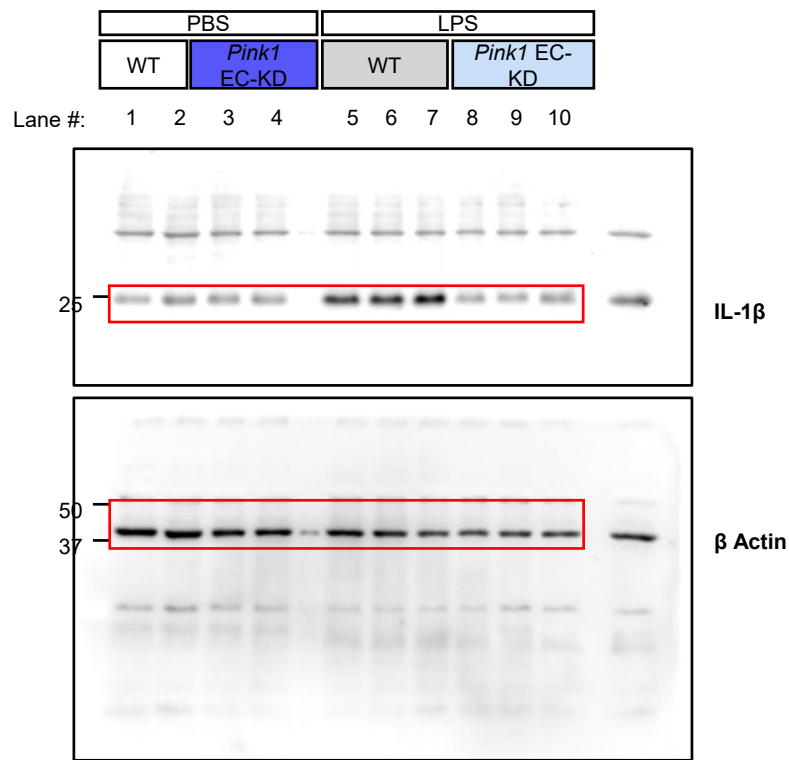

Supplement: Figure 4—source data 2. [file elife-82205-fig4-data2.pdf]

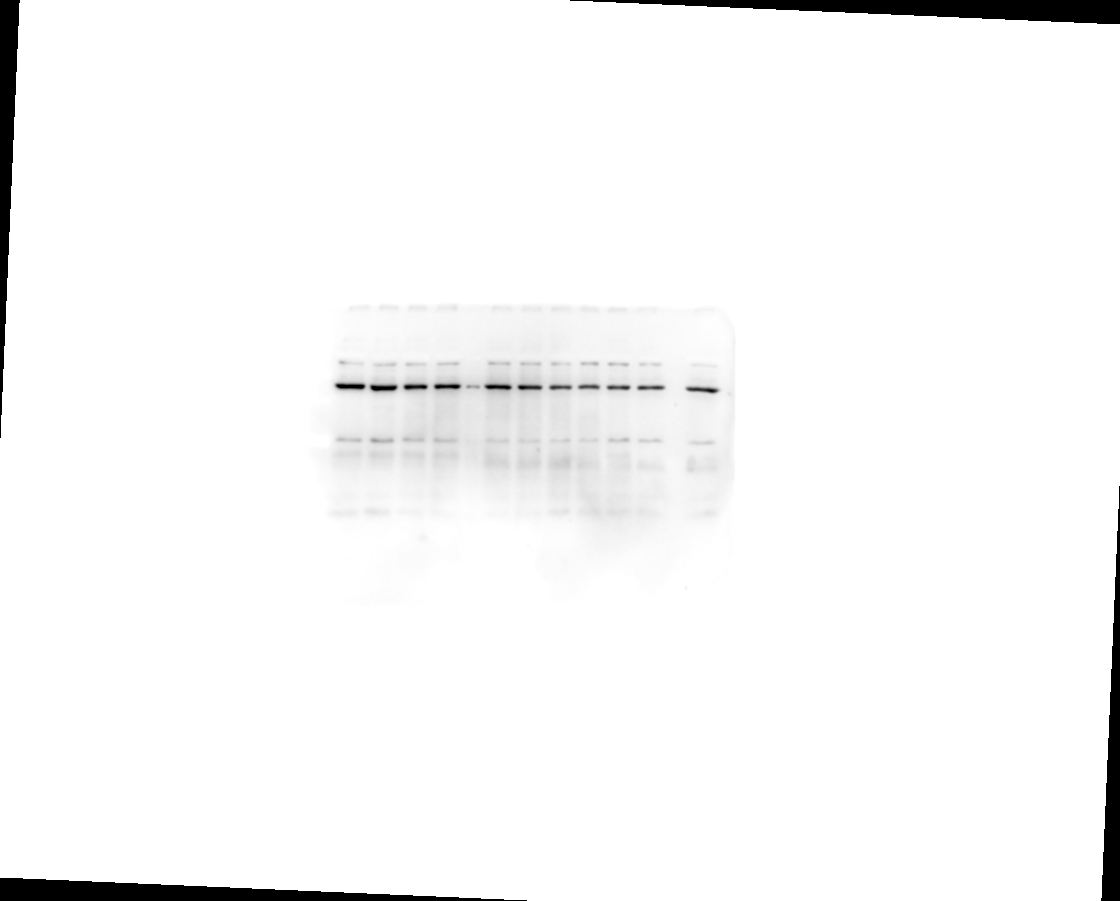

Supplement: Figure 4—source data 3. [file elife-82205-fig4-data3.zip › Figure 4 Source Data 3/Figure_4F_Beta_Actin.tif]

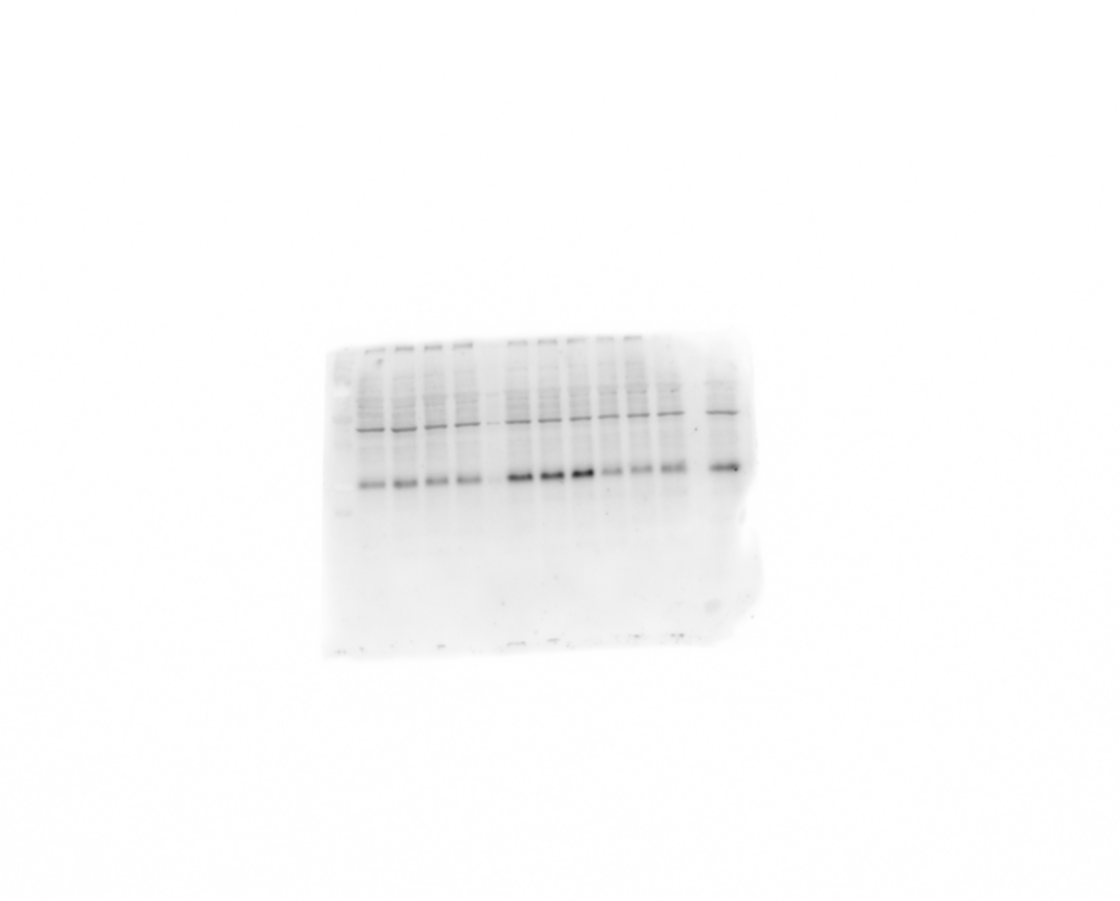

Supplement: Figure 4—source data 3. [file elife-82205-fig4-data3.zip › Figure 4 Source Data 3/Figure_4F_IL1B.tif]

Figure 4, figure supplement 1 B

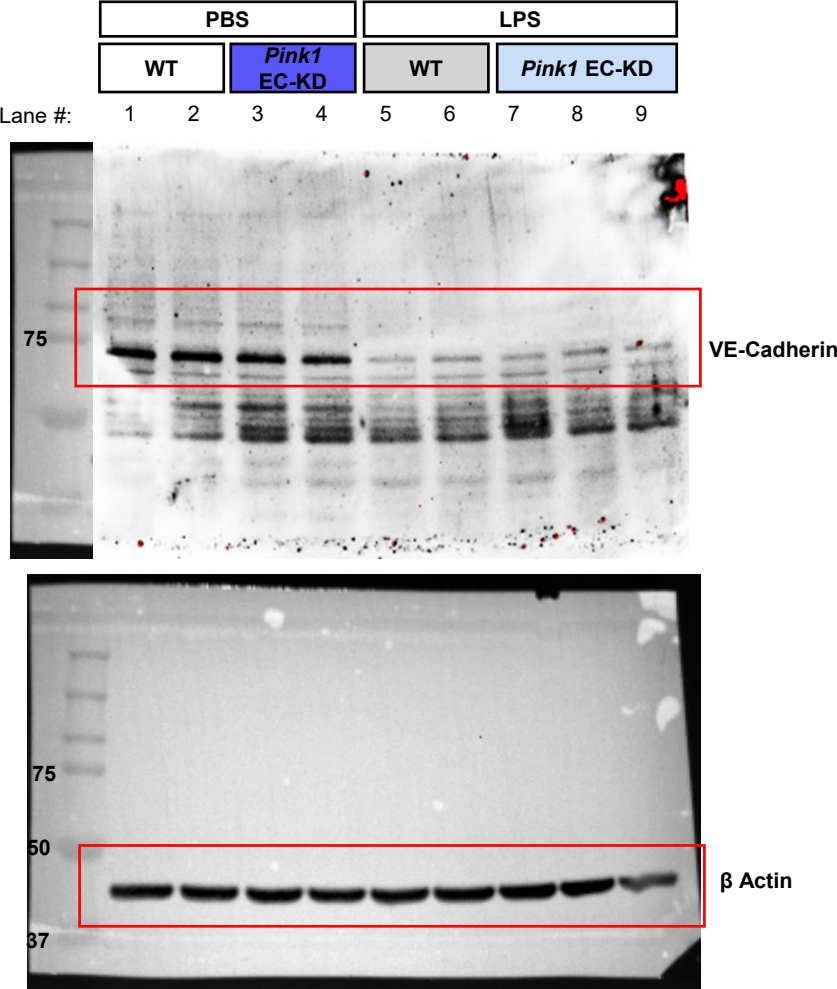

Supplement: Figure 4—figure supplement 1—source data 2. [file elife-82205-fig4-figsupp1-data2.pdf]

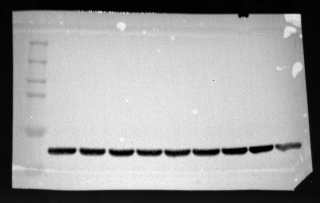

Supplement: Figure 4—figure supplement 1—source data 3. [file elife-82205-fig4-figsupp1-data3.zip › Figure 4 figure supplement 1 source data 3/Figure_4_Supplement_1B_ Beta_Actin.tif]

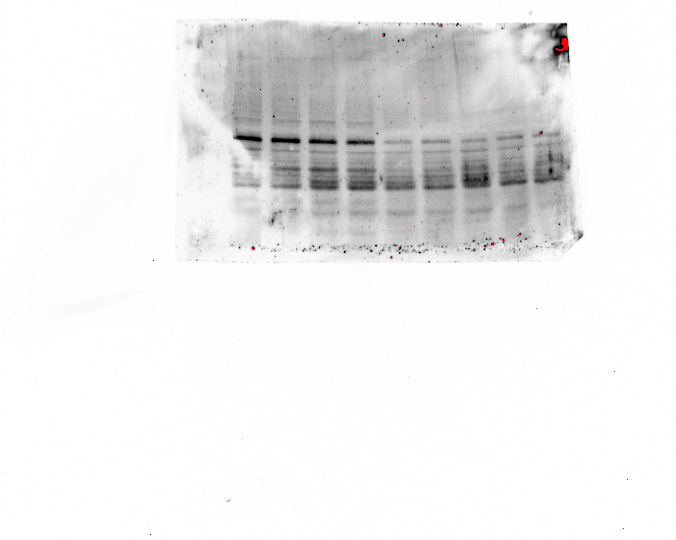

Supplement: Figure 4—figure supplement 1—source data 3. [file elife-82205-fig4-figsupp1-data3.zip › Figure 4 figure supplement 1 source data 3/Figure_4_Supplement_1B_VE_Cadherin.tif]

Figure 5 B:

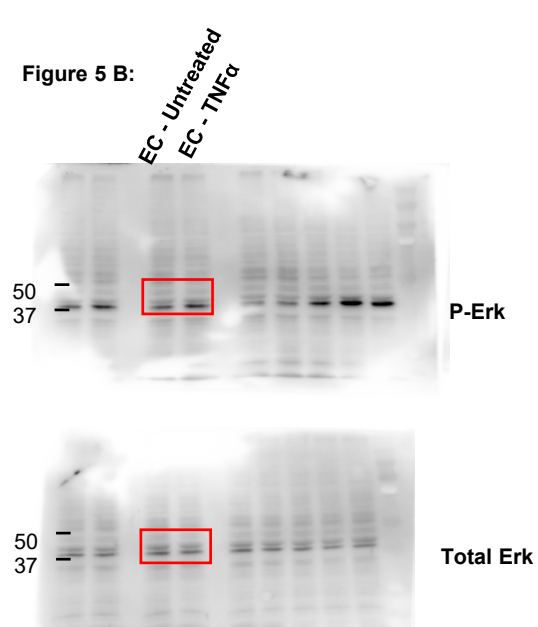

Figure 5 E:

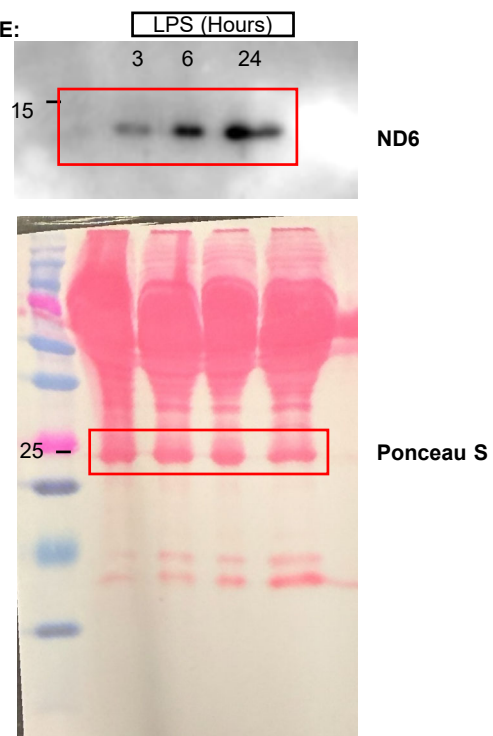

Figure 5 G:

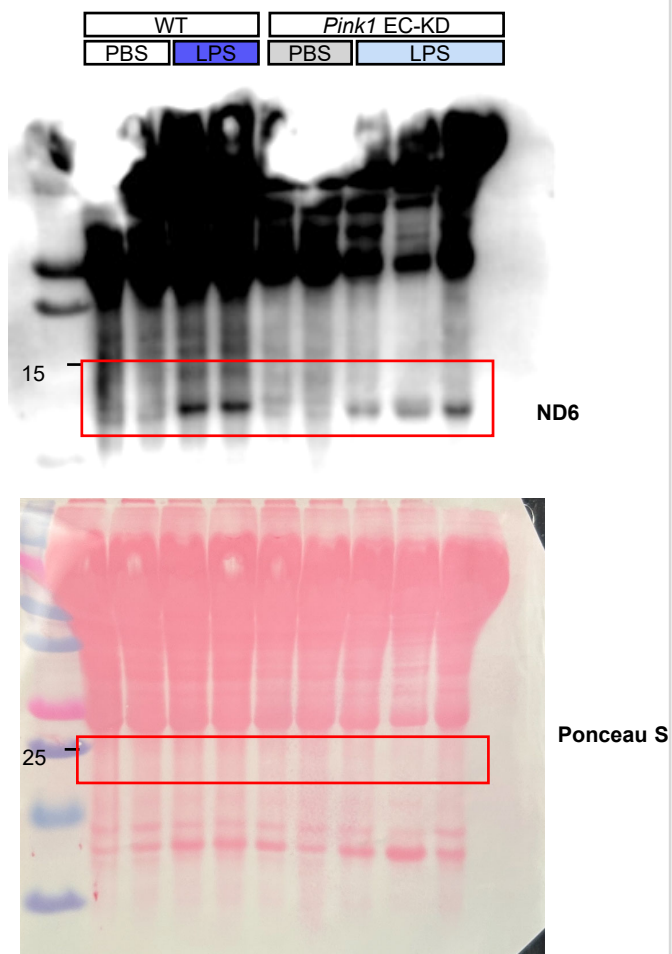

Supplement: Figure 5—source data 2. [file elife-82205-fig5-data2.pdf]

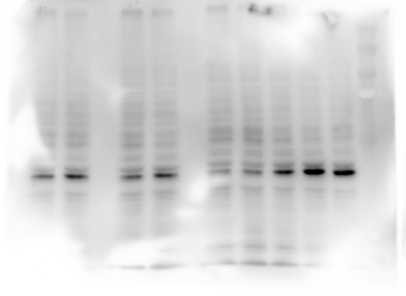

Supplement: Figure 5—source data 3. [file elife-82205-fig5-data3.zip › Figure 5 Source Data 3/Figure_5B_Phospho_Erk.tif]

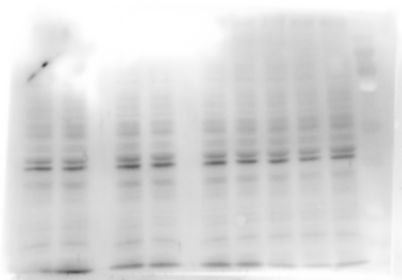

Supplement: Figure 5—source data 3. [file elife-82205-fig5-data3.zip › Figure 5 Source Data 3/Figure_5B_Total_Erk.tif]

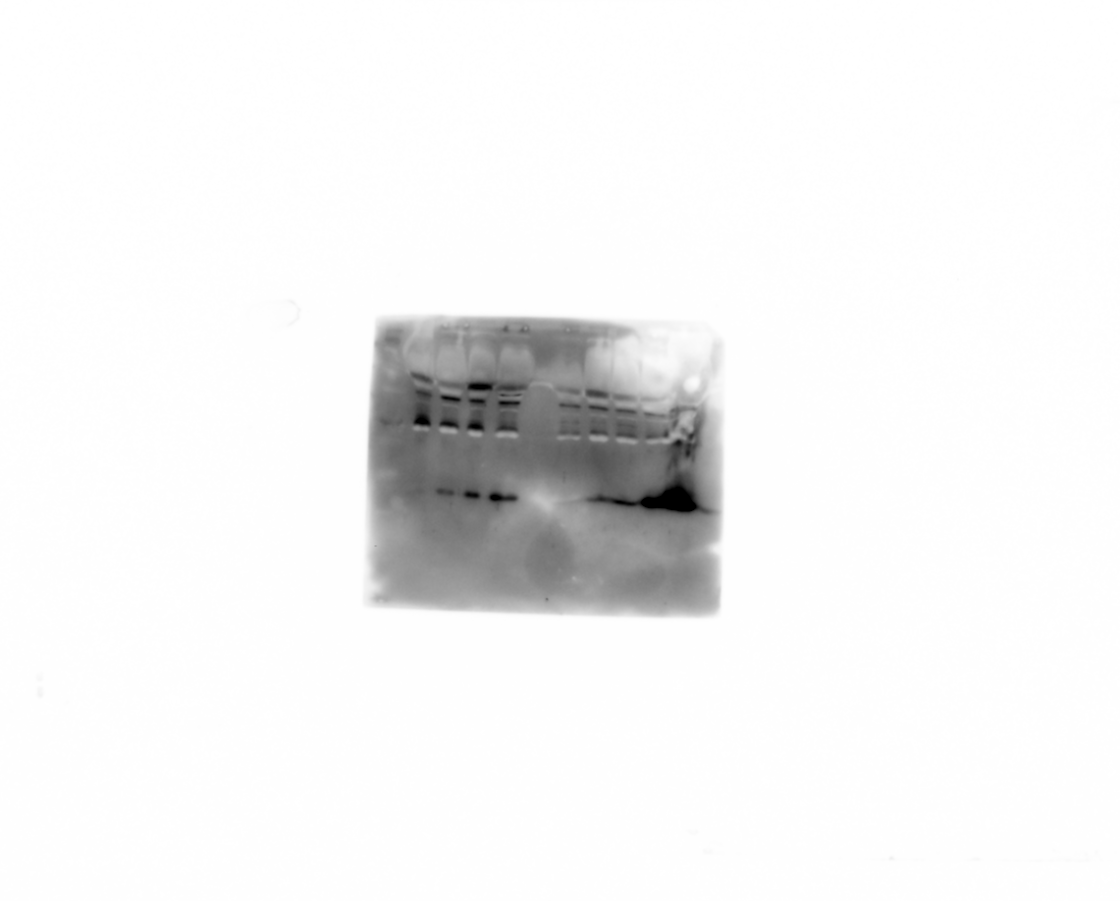

Supplement: Figure 5—source data 3. [file elife-82205-fig5-data3.zip › Figure 5 Source Data 3/Figure_5E_ND6.tif]

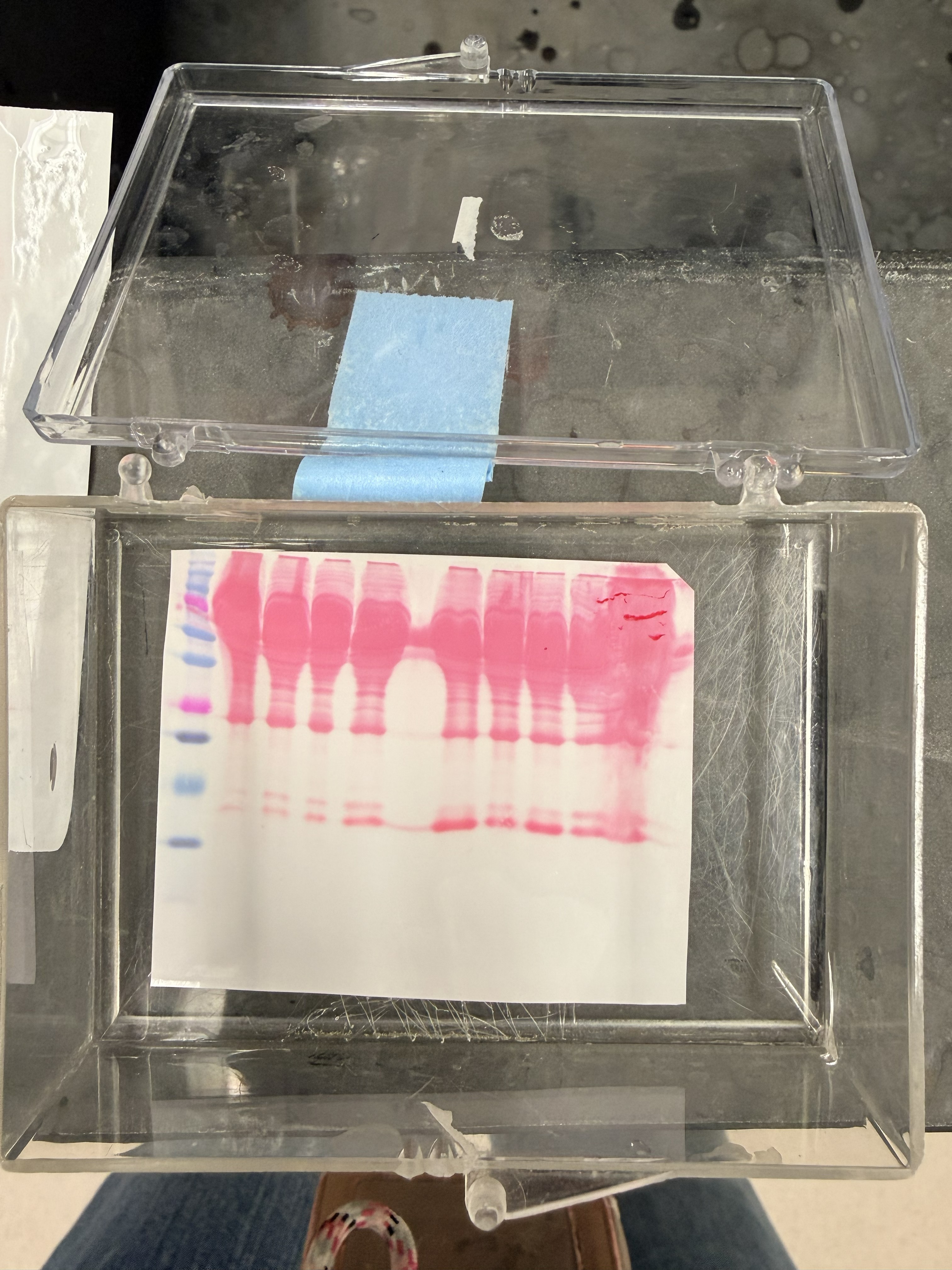

Supplement: Figure 5—source data 3. [file elife-82205-fig5-data3.zip › Figure 5 Source Data 3/Figure_5E_Ponceau.jpg]

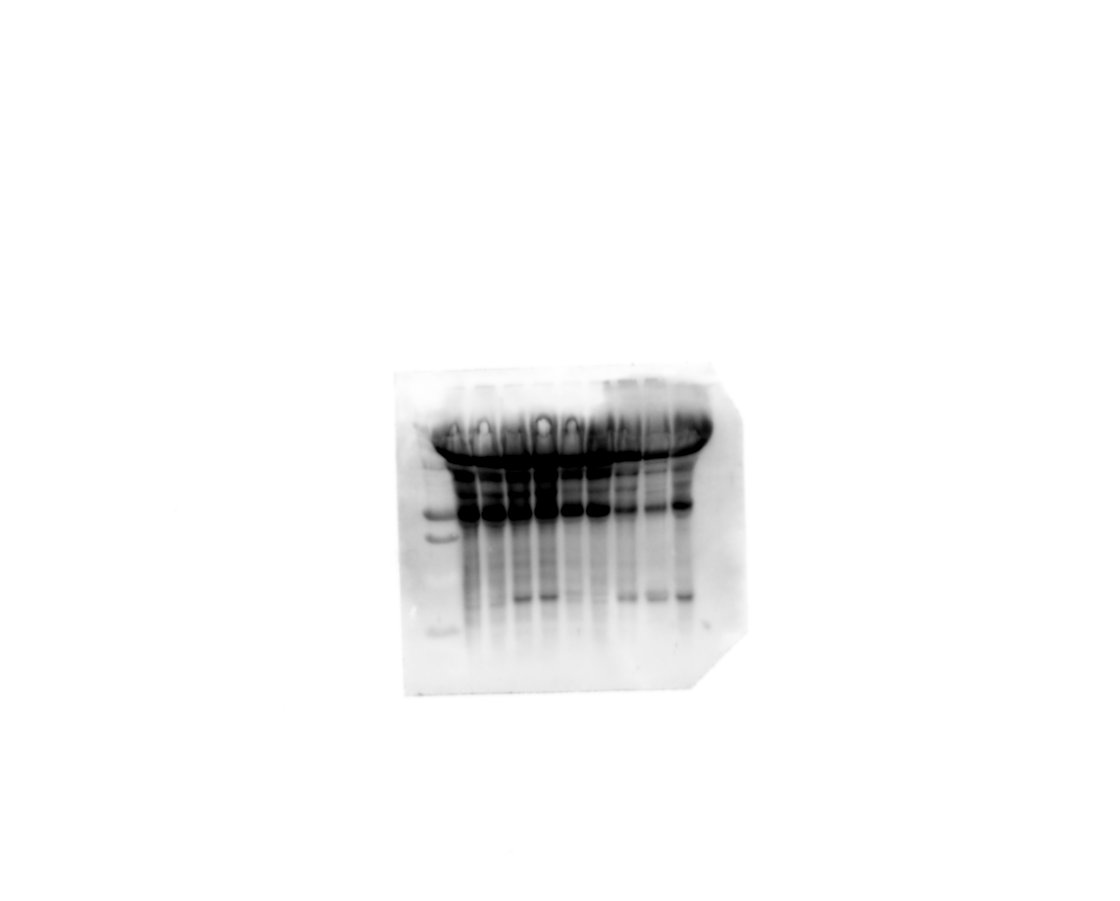

Supplement: Figure 5—source data 3. [file elife-82205-fig5-data3.zip › Figure 5 Source Data 3/Figure_5G_ND6.jpg]

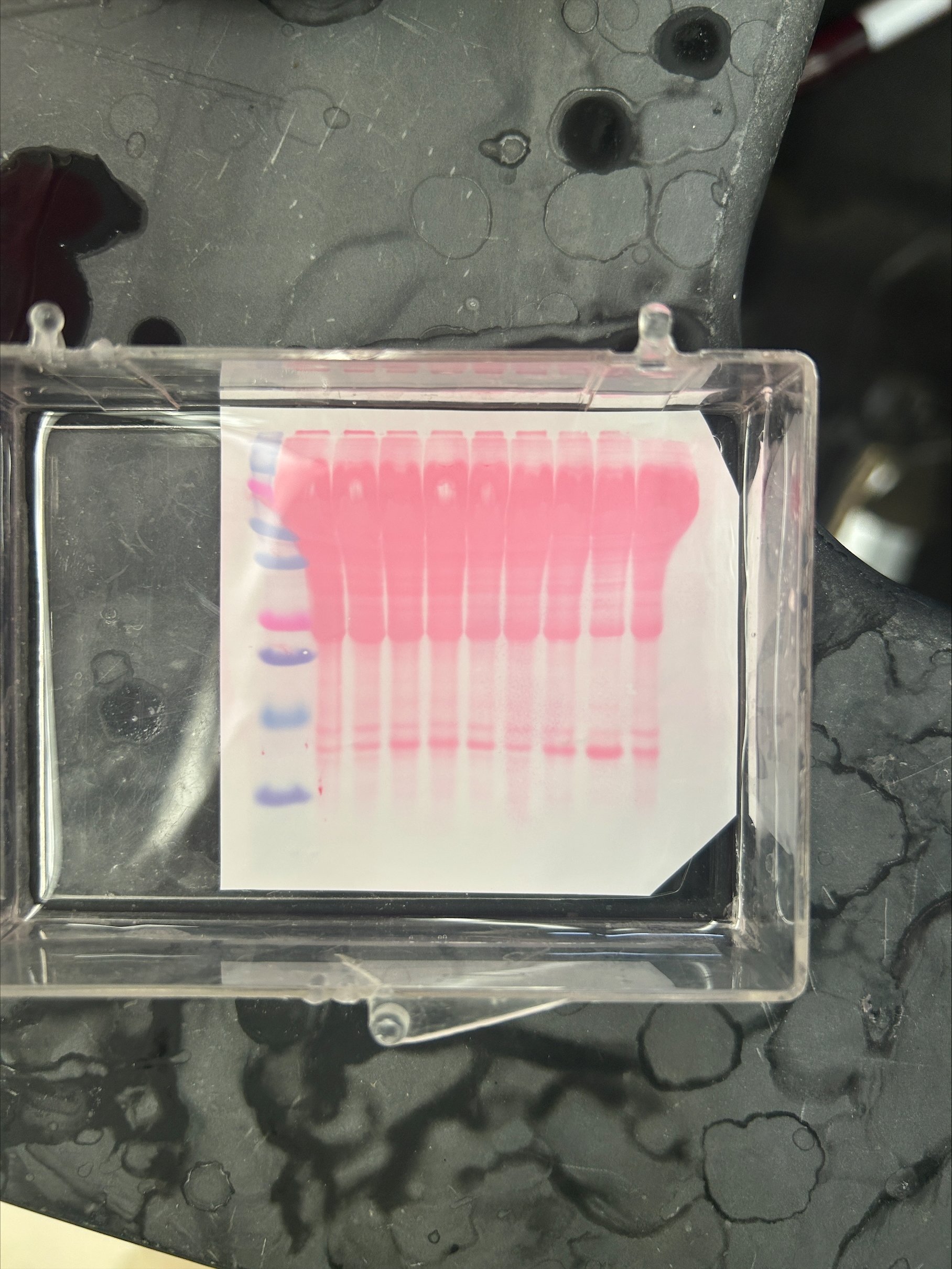

Supplement: Figure 5—source data 3. [file elife-82205-fig5-data3.zip › Figure 5 Source Data 3/Figure_5G_Ponceau.jpg]

Figure 5, figure supplement 1 B:

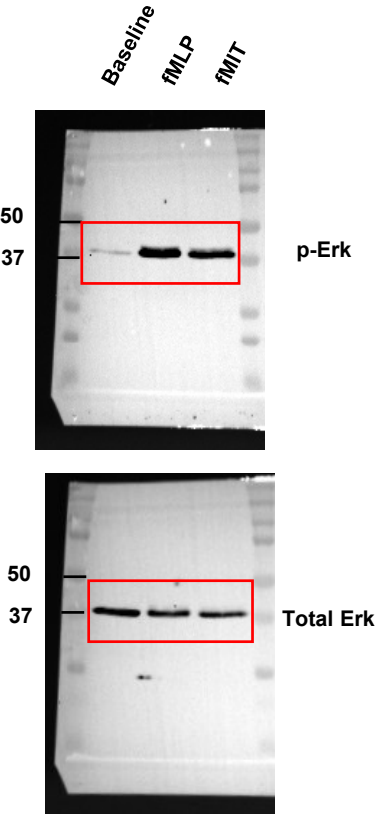

Supplement: Figure 5—figure supplement 1—source data 2. [file elife-82205-fig5-figsupp1-data2.pdf]

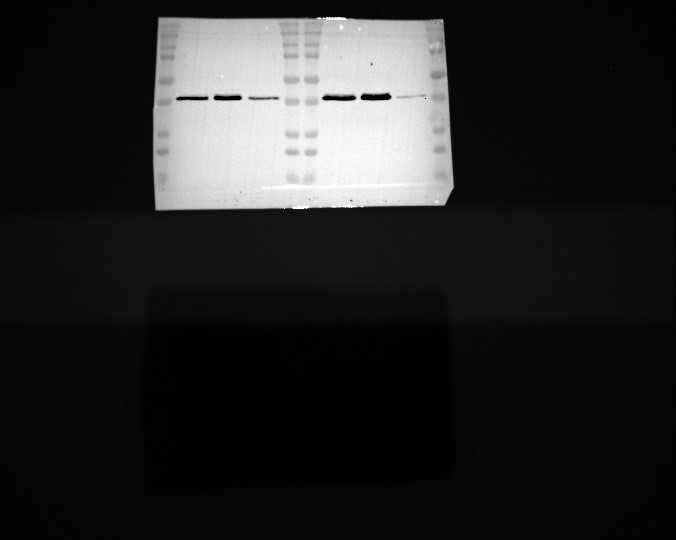

Supplement: Figure 5—figure supplement 1—source data 3. [file elife-82205-fig5-figsupp1-data3.zip › Figure 5 figure supplement 1 source data 3/Figure_5_Supplement_1B_Phospho_Erk.jpg]

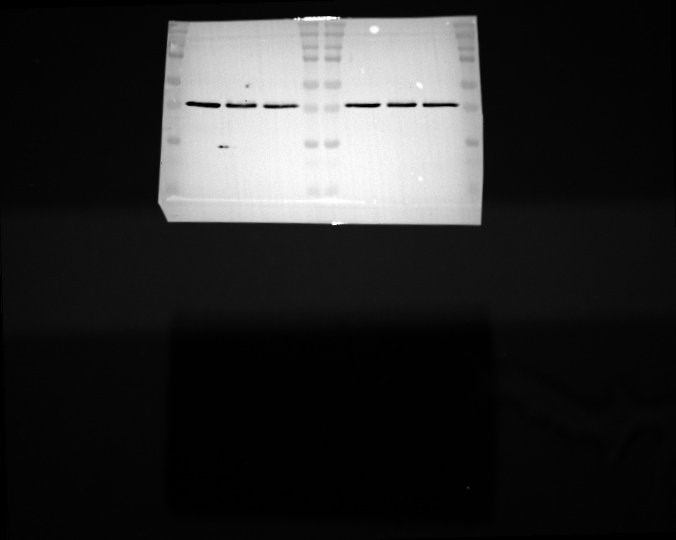

Supplement: Figure 5—figure supplement 1—source data 3. [file elife-82205-fig5-figsupp1-data3.zip › Figure 5 figure supplement 1 source data 3/Figure_5_Supplement_1B_Total_Erk.jpg]
