# Supplementary material for: Pink1-mediated mitophagy in the endothelium releases proteins encoded by mitochondrial DNA and activates neutrophil responses during inflammation
Source: eLife. 2026 Jul 1;15:e82205. doi: 10.7554/eLife.82205 (PMC13423353; doi:10.7554/eLife.82205)
Supplement: Peer review history

# Editor report: Editor's evaluation

**DOI:** 10.7554/eLife.82205.sa0

**Authors:** Noboru Mizushima

**Affiliations:** https://ror.org/057zh3y96 The University of Tokyo Japan

The authors propose that TNFα induces mitochondrial depolarization and PINK1 recruitment, and that PINK1-dependent endothelial mitophagy promotes inflammation by increasing the release of mitochondrial formyl peptides, a class of mitochondrial DAMPs. Both the in vivo and in vitro data are convincing. This fundamental study identifies a potential source of circulating formyl peptides during inflammation and reveals a novel pro-inflammatory role for mitophagy, which may be context- and/or tissue-specific.

# Decision letter

**DOI:** 10.7554/eLife.82205.sa1

**Affiliations:** https://ror.org/057zh3y96 The University of Tokyo Japan; https://ror.org/057zh3y96 The University of Tokyo Japan

Our editorial process produces two outputs: (i) public reviews designed to be posted alongside the preprint for the benefit of readers; (ii) feedback on the manuscript for the authors, including requests for revisions, shown below. We also include an acceptance summary that explains what the editors found interesting or important about the work.

**Decision letter after peer review:**

Thank you for submitting your article "Pink1-mediated mitophagy in the endothelium releases proteins encoded by mitochondrial DNA and activates neutrophil responses" for consideration by *eLife*. Your article has been reviewed by 3 peer reviewers, and the evaluation has been overseen by a Reviewing Editor and Carla Rothlin as the Senior Editor. The following individual involved in review of your submission has agreed to reveal their identity: Noboru Mizushima (Reviewer #1).

The reviewers have discussed their reviews with one another, and the Reviewing Editor has drafted this to help you prepare a revised submission.

*Essential Revisions:*

1. Evidence of PINK1 activation by TNFa is not enough.

- Does TNFa induce Parkin recruitment to mitochondrial, ubiquitin phosphorylation, and/or mitochondrial depolarization? (Reviewer #2-3, Revier #3-Conceptual 1)

2. Requirement of PINK1 in mitophagy and ND6 release should be investigated.

- Does loss of PINK1 block TNFa-induced mitophagy in endothelial cells (Reviewer #2-5)?

- Figure 4. Does loss of PINK1 block LPS-induced endothelial mitophagy in mitoKeima mouse model (Reviewer #2-7)?

- Figure 6. Does loss of PINK1 alter ND6 release in response to TNF/FCCP in vitro (Reviewer #2-9, Reviewer #3-Conceptual 4) and to LPS in vivo (Reviewer #1)?

3. Requirement of lysosomal degradation should be investigated.

- Is lysosomal hydrolysis important for ND6 release? This can be tested by using lysosomal inhibitors such as bafilomycin A1 (Reviewer #2-10).

4. Other technical issues

- Figure 4. The knockdown efficiency of PINK1 is not robust. Please confirm it with downstream markers such as the phosphorylation of Parkin or ubiquitin or the phosphorylation of Rab8 S111 (Reviewer #2-6).

- Keima is acidification-dependent (i.e., ATP-dependent). Describe the time required to prep and image these mouse lungs (Reviewer #3-Technical 1).

- Describe more details about quantification and statistical analysis (Reviewer #3-Technical 1, 2).

*Reviewer #1 (Recommendations for the authors):*

1. The authors clearly show that mitophagy induces ND6 secretion from endothelial cells in vitro. Also, they hypothesize that majority of serum formyl peptides are derived from endothelial cells during inflammation. However, this is not tested in vivo, even though the authors show that endothelial-specific Pink1-knockout mice exhibit improved inflammation after LPS treatment. To support the authors' hypothesis that endothelial mitophagy is indeed the origin of serum formyl peptides, it would be critical to measure the serum ND6 levels in wild-type and Pink1-KO mice before and after LPS treatment.

*Reviewer #2 (Recommendations for the authors):*

The authors certainly have an interesting set of results that could be impactful for the mitophagy/inflammation/endothelial biology field. However, as mentioned in the "public review" the link between their results needs to be strengthened and at the moment appear somewhat preliminary. As discussed below, Is PINK1 really activated and if so, are there any insights into the mechanism? Does loss of PINK1 in cells and tissues block LPS/TNFa induced mitophagy? While the major role of PINK1 currently identified is in regulating mitophagy, it has been shown to be important in other processes too (see Pollock et al., Bioessays 2021. PMID: 34617288).

Main Points:

1) In Figure 2A-B, it would be beneficial to have a positive control. For example, how does TNFa mitophagy compare to that induced by mitochondrial depolarisation?

2) In Figure 2D, I am not sure what the significance of this observation is. The red-only structures are lysosomal, and they appear to make contact with mitochondria. As it is already known that lysosomes can contact mitochondria, is there an additional point here? Is the contact with lysosomes different with TNFa treatment? Are they newly formed mitolysosomes, or pre-existing "red" structures?

3) The data in Figure 3 for PINK1 activation needs to be strengthened. Does TNF increase phospho-ubiquitin levels? Does it lead to Parkin recruitment to mitochondria? How do levels compare with a positive control such as mitochondrial depolarization?

4) Related, how is TNFa activating PINK1? Are mitochondria depolarized? Is import blocked?

5) Also related, does loss of PINK1 block TNFa-induced mitophagy in the endothelial cells?

6) In Figure 4, the level of PINK1 depletion does not look very high, especially given the loading control is also less in these lanes. These data should be quantified. Of note, PINK1 is notoriously difficult to detect by western blot in mouse tissues, especially with commercial antibodies. The authors could also consider a downstream protein marker.

7) Also in relation to Figure 4, does loss of PINK1 block LPS-induced endothelial mitophagy in their mitoKeima mouse model?

8) In Figure 5, how does the temporal response to LPS relate to mitophagy induction? Some discussion at least would be helpful.

9) In Figure 6 A-B, does loss of PINK1 alter ND6 release in response to TNFa/FCCP?

10) Related to the mechanism of ND6 release, is it impaired by inhibition of lysosomal hydrolysis (for example with Bafilomycin A1)?

11) In Figure 6C-D, a non-formylated peptide control is also needed.

12) In Figure 6E-G, ERK activation does necessarily mean an inflammatory response. Could the authors monitor some downstream markers such as cytokine release and if so, is this blocked by MEK/ERK inhibitors?

*Reviewer #3 (Recommendations for the authors):*

To establish this point, the authors need to demonstrate PINK1-dependent secretion of mitochondria-derived peptides after LPS addition in vivo. They also need to show an essential role for fMLP receptors on neutrophils in vivo. In its present state, the manuscript is inconclusive.

Technical criticisms

Regarding figure 1, as mitophagy is a very dynamic process and the Keima fluorophore is very sensitive to environmental conditions, I think that the authors should report the time required to prep and image these mouse lungs.

The quantification of in vivo mitophagy for figure 1 reports four animals per treatment condition, from what I assume was representative of three experiments. I struggle to understand how the authors could have normalized all PBS-treated datapoints to 1.0. It would be much better if the authors could report something simple with biological variation such as a mito-lysosomal area or intensity.

Similar to comment #2, the method of quantification for Figure 2B seems incorrect. The authors appear to be reporting variation in mitophagy between imaging fields. Please report the data across replicate experiments.

Regarding Figure 2c-d, the authors show an example of prolonged contact between a mitochondrion and a mito-lysosome. Without an untreated control and quantification, it is unconvincing that this is remarkable and/or unique to TNFa treatment.

Figure 5e, the authors need to state the timepoint used for this blot.

Figure 6 could also be supplemented with TEM to observe the ultrastructural of the TNFa-induced mitophagosomes, +/- PINK1 knockout.

A number of conclusions are based on statistically underpowered figures. In Figure 4S1A and S2, and 5S1B, there are effects of similar or larger magnitude compared to other panels, but they do not reach significance because of small n.

# Reply: Author response

**DOI:** 10.7554/eLife.82205.sa2

Essential Revisions:

1. Evidence of PINK1 activation by TNFa is not enough.

- Does TNFa induce Parkin recruitment to mitochondrial, ubiquitin phosphorylation, and/or mitochondrial depolarization? (Reviewer #2-3, Revier #3-Conceptual 1)

We agree that providing additional evidence for TNFα activating the Pink1 pathway would be helpful. We previously showed that TNFα stabilizes Pink1 in endothelial cells by evaluating Pink1 protein levels via immunoblotting (originally Figure 3A-B, now moved to Figure 2, figure supplement 2A-B). To determine whether this stabilized Pink1 was involved in mitophagy, we now examined whether TNFα induces mitochondrial localization of Pink1.

Human lung microvascular endothelial cells (HLMVECs) were treated with either PBS or TNFα (10 ng/mL) for 3 hours, fixed and then immuno-stained for Pink1 and the mitochondrial outer membrane protein Tomm20. Mitochondria and Pink1 were visualized by confocal microscopy. Consistent with our immunoblotting data, total Pink1 levels, calculated as Pink1 intensity normalized to Tomm20 intensity, increased in response to TNFα (new Figure 2, figure supplement 3).

We created z-stack reconstructions of these images to more rigorously assess the mitochondrial localization and determined that Pink1 was localized to Tomm20-stained mitochondria (new Figure 2C). Arrows indicate areas of colocalization of Pink1 with Tomm20.

Mitochondrial Pink1 was visualized by creating a mitochondrial mask based on the mitochondrial membrane protein Tomm20, and we applied this mask to images of Pink1 (new Figure 2D). We determined the proportion of mitochondria containing Pink1 by calculating the Manders’ coefficient Tomm20 showing co-localization with Pink1. In response to TNFα, we observed a 5fold increase in the proportion of Tomm20 pixels overlapping with Pink1 (new Figure 2E). We next quantified the total amount of Pink1 localized to the mitochondrial compartment. The total intensity of mitochondrial Pink1 was normalized to the intensity of Tomm20, to account for changes in mitochondrial content per visual field. The average mitochondrial Pink1/Tomm20 over all visual fields in each replicate experiment was plotted. Compared to untreated controls, endothelial cells treated with TNFα had a 6-fold increase in mitochondrial Pink1 (new Figure 2F). These new data provide quantitative evidence for TNFα-induced recruitment of Pink1 to the mitochondria.

Furthermore, in our revision we additionally now demonstrate that TNFα induces rapid mitochondrial depolarization which is a well-established mechanism for activating Pink1 and mitophagy (Figure 2, figure supplement 1A-C).

2. Requirement of PINK1 in mitophagy and ND6 release should be investigated.

- Does loss of PINK1 block TNFa-induced mitophagy in endothelial cells (Reviewer #2-5)?

- Figure 4. Does loss of PINK1 block LPS-induced endothelial mitophagy in mitoKeima mouse model (Reviewer #2-7)?

We thank the reviewers for this comment, and we agree that it is important to determine whether endothelial Pink1 is required for LPS-induced mitophagy. Because it was not possible to cross our Pink1^EC-/-^ mouse with the Mitokeima mouse due to spectral overlap of the fluorescence, we examined mitophagy using colocalization of immunofluorescent staining for the lysosomal marker LAMP1 and the mitochondrial marker Tomm20 in control and Pink1^EC-/-^ mouse lung sections. Mice were injected with either LPS (8 mg/kg) or PBS, and lungs were harvested after 6 hours. Lungs were fixed in 4% paraformaldehyde (PFA), embedded in Optimal Cutting Temperature (OCT) and cryo-sectioned before staining against LAMP1, Tomm20 and DAPI. LPS induced a significant increase in colocalization of Tomm20-stained mitochondria and LAMP1-stained lysosomes, indicating an increase in lysosome-engulfed mitochondria, and thus mitophagy (new Figure 3CD). Compared to control mice, Pink1^EC-/-^ mice had significantly reduced LPS-induced mitophagy, and in fact had no induction of mitophagy compared to PBS-injected counterparts. Our findings thus suggest that endothelial Pink1 is indeed necessary for LPS-induced endothelial mitophagy.

- Figure 6. Does loss of PINK1 alter ND6 release in response to TNF/FCCP in vitro (Reviewer #2-9, Reviewer #3-Conceptual 4) and to LPS in vivo (Reviewer #1)?

We thank the reviewer for this important suggestion and agree that addressing the role of Pink1 for the release of ND6 is important. To examine this question, we first determined whether inflammation-induced ND6 release also occurs in vivo. We reasoned that studying this question in a mouse model would allow for a temporal comparison to the observed phenotypic changes in neutrophil recruitment (as seen in original Figure 5A-D, current Figure 4B-E), as well as determine whether endothelial cells are a substantial source of ND6 release in vivo.

We collected blood plasma from wildtype C57/bl6 mice injected with LPS (8 mg/kg), or PBS as a control, and evaluated plasma ND6 levels over time by immunoblotting. As seen in the new Figure 5E-F, plasma ND6 levels were significantly increased at 6 hours following LPS injection, which were further increased at 24 hours.

Next, to study the link between endothelial Pink1 and ND6 release, we evaluated plasma ND6 levels in Pink1^EC-/-^ and control mice that had been injected with LPS (8 mg/kg). We found that compared to controls, Pink1^EC-/-^ mice had significantly reduced plasma ND6 (new Figure 5G-H). This finding not only links endothelial Pink1 to the inflammatory release of ND6 but also suggests that endothelial cells are a significant source of circulating ND6 in response to inflammation.

3. Requirement of lysosomal degradation should be investigated.

- Is lysosomal hydrolysis important for ND6 release? This can be tested by using lysosomal inhibitors such as bafilomycin A1 (Reviewer #2-10).

We thank the reviewer for bringing up this intriguing question, particularly given recent findings that lysosomal compromise triggers extracellular mitochondrial release(Liang, Sagar et al. 2023). The role of lysosomal biology in release of mitochondrial proteins constitutes a fundamental cell biology question. As our manuscript focuses on the inflammatory responses and neutrophil recruitment, we feel that such a lysosomal project really merits a separate manuscript. We acknowledge the importance of this question in the revised discussion.

4. Other technical issues

- Figure 4. The knockdown efficiency of PINK1 is not robust. Please confirm it with downstream markers such as the phosphorylation of Parkin or ubiquitin or the phosphorylation of Rab8 S111 (Reviewer #2-6).

We agree that it would be helpful to provide additional evidence for successful Pink1 depletion and develop more robust and consistent lung endothelial Pink1 depletion as this would be of value to readers. As Pink1 depletion is a key method used in our manuscript, we further optimized the Pink1 depletion in vivo by applying nanoparticle delivery of Pink1 sgRNA to VECad-Cas9 mice to induce in vivo CRISPR deletion of Pink1 in endothelial cells. We created zwitterionic vesicles to encapsulate purified control or Pink1-sgRNA, which were introduced to mice intranasally, and knockdown efficiency in endothelial cells evaluated by immunoblotting.

Using this novel delivery system, we were able to achieve robust knockdown of endothelial Pink1. Given that Pink1 protein levels are suppressed by degradation under homeostatic conditions, but can be rapidly increased by loss of degradation, we additionally examined whether residual amounts of Pink1 in our knockdown model could be stabilized by LPS to near-endogenous levels. Although Pink1 levels increased in control mice injected with 8 mg/kg LPS, they remained lowered in Pink1^EC-/-^ mice, with no significant increase over PBS-injected controls (new Figure 3A-B).

The methods for nanoparticle generation and delivery have now been included in the revised Results section of the manuscript. The delivery system used in each experiment has been noted in all figure legends.

- Keima is acidification-dependent (i.e., ATP-dependent). Describe the time required to prep and image these mouse lungs (Reviewer #3-Technical 1).

We thank the reviewer for bringing this oversight to our attention. We have expanded on the details of the method used both in the results and methods sections.

- Describe more details about quantification and statistical analysis (Reviewer #3-Technical 1, 2).

Additional details describing the quantification and statistical analysis have been added to the methods section and figure legends.

Reviewer #1 (Recommendations for the authors):

1. The authors clearly show that mitophagy induces ND6 secretion from endothelial cells in vitro. Also, they hypothesize that majority of serum formyl peptides are derived from endothelial cells during inflammation. However, this is not tested in vivo, even though the authors show that endothelial-specific Pink1-knockout mice exhibit improved inflammation after LPS treatment. To support the authors' hypothesis that endothelial mitophagy is indeed the origin of serum formyl peptides, it would be critical to measure the serum ND6 levels in wild-type and Pink1-KO mice before and after LPS treatment.

We thank the reviewer for the excellent suggestion to test whether ND6 is released in vivo. The addition of these experiments has greatly improved the comprehensiveness of this study. We examined the presence of ND6 in mouse plasma samples using western blot and found that ND6 is indeed released into circulation in response to LPS-induced inflammation within 6 hours (new Figure 5E-F).

Furthermore, we found that deletion of endothelial Pink1 significantly decreases plasma ND6 levels following LPS (new Figure 5G-H).

These results suggest that circulating ND6 is dependent on endothelial Pink1. Combined with our in vitro data which indicated that endothelial cells release ND6 into the cell culture medium in response to TNFα (originally Figure 6B, now moved to Figure 5D), these data suggest that endothelial cells are a significant source of serum ND6.

Reviewer #2 (Recommendations for the authors):

The authors certainly have an interesting set of results that could be impactful for the mitophagy/inflammation/endothelial biology field. However, as mentioned in the "public review" the link between their results needs to be strengthened and at the moment appear somewhat preliminary. As discussed below, Is PINK1 really activated and if so, are there any insights into the mechanism? Does loss of PINK1 in cells and tissues block LPS/TNFa induced mitophagy? While the major role of PINK1 currently identified is in regulating mitophagy, it has been shown to be important in other processes too (see Pollock et al., Bioessays 2021. PMID: 34617288).

Main Points:

1) In Figure 2A-B, it would be beneficial to have a positive control. For example, how does TNFa mitophagy compare to that induced by mitochondrial depolarisation?

As a positive control, we used a cocktail of Oligomycin/Antimycin A (O/A) to induce mitochondrial depolarization. We observed that TNFα and O/A induced similar levels of mitophagy as measured by mitokeima (data presented here for reviewers only, as this has been published by several groups and is an established control experiment).

**Author response image 1.**

2) In Figure 2D, I am not sure what the significance of this observation is. The red-only structures are lysosomal, and they appear to make contact with mitochondria. As it is already known that lysosomes can contact mitochondria, is there an additional point here? Is the contact with lysosomes different with TNFa treatment? Are they newly formed mitolysosomes, or pre-existing "red" structures?

We agree and have removed it from the revised manuscript.

3) The data in Figure 3 for PINK1 activation needs to be strengthened. Does TNF increase phospho-ubiquitin levels? Does it lead to Parkin recruitment to mitochondria? How do levels compare with a positive control such as mitochondrial depolarization?

We appreciate this comment and the need to further examine Pink1 activation. To add to our finding of Pink1 stabilization from the original manuscript, we additionally probed whether Pink1 is also localized to the mitochondria in response to TNFα. This was done by immunostaining Pink1 and the mitochondrial marker Tomm20 in cultured endothelial cells (new Figure 2C-D). We observed that in response to TNFα, the proportion of Tomm20 overlapped with Pink1 increased 5-fold, indicating that more mitochondria are associated with Pink1(new Figure 2E). Additionally, we observed a significant increase in the total amount of Pink1 that was mitochondrially localized (new Figure 2F).

4) Related, how is TNFa activating PINK1? Are mitochondria depolarized? Is import blocked?

We thank the reviewer for this comment. We addressed whether TNFα altered mitochondrial potential using the cationic dye Tetramethylrhodamine Methyl Ester (TMRM). HLMVECs were treated with TNFα over 1 hour time course and stained with TMRM 30 minutes prior to imaging. We found that the addition of TNFα led to significant mitochondrial depolarization within 5 minutes, and persisting up to 1 hour (new Figure 2, figure supplement 1).

5) Also related, does loss of PINK1 block TNFa-induced mitophagy in the endothelial cells?

We appreciate this comment and agree that it is important to assess whether Pink1 is required for inflammation-mediated mitophagy. We opted to test this in vivo using our Pink1^EC-/-^ mice. Control and Pink1^EC-/-^ mice were injected with LPS, and at the 6 hours post LPS time point, lungs were harvested, fixed and sectioned. Lung sections were immuno-stained for Tomm20 to label the mitochondria, and Lamp1 to label lysosomes. Mitophagy was measured as the overlap of Tomm20 and Lamp1, indicating mitochondria localized in the lysosome. As observed using mitoKeima, Mitolysosome formation (indicator of mitophagy) increased in response to LPS.

Importantly, the deletion of Pink1 prevented mitophagy from occurring above basal levels (new Figure 3C-D). This data indicates that inflammation-mediated endothelial mitophagy is dependent on Pink1.

6) In Figure 4, the level of PINK1 depletion does not look very high, especially given the loading control is also less in these lanes. These data should be quantified. Of note, PINK1 is notoriously difficult to detect by western blot in mouse tissues, especially with commercial antibodies. The authors could also consider a downstream protein marker.

We are grateful for this comment, and the original Figure 4B-E has now been moved to Figure 3, figure supplement 2. We addressed this comment by including a new figure in which we assess Pink1 in the presence of LPS treatment because Pink1 levels could be influenced by inflammation and therefore inflammatory upregulation compensates for some of the PINK1 depletion (new Figure 3A-B).

This is why the reviewer’s suggestion to examine additional downstream markers of autophagy was very helpful. We examined conversion of the autophagy marker LC3, which is involved in initiation of the autophagosome. We found that LPS stimulated increased conversion of LC3b in endothelial cells, but that this conversion was abrogated by Pink1 depletion (new Figure 3E-F). This further underscores that Pink1 depletion indeed leads to reduced autophagy in the endothelium.

7) Also in relation to Figure 4, does loss of PINK1 block LPS-induced endothelial mitophagy in their mitoKeima mouse model?

As discussed in response #5, loss of Pink1 does indeed block LPS-induced endothelial mitophagy (new Figure 3C-D).

8) In Figure 5, how does the temporal response to LPS relate to mitophagy induction? Some discussion at least would be helpful.

In response, we highlight the time points in the text of the Results section for Figure 4. Briefly, our revised results establish 6 hours following LPS administration as a key time point linking three phenotypes which are sensitive to endothelial Pink1 deletion. The first is the induction of mitophagy, which occurs within 6 hours and is significantly lowered in Pink1^EC-/-^ mice (new Figure 3C-D). The second is neutrophil recruitment to the lung, which is reduced in Pink1^EC-/-^ mice (originally Figure 5A, now moved to Figure 4B). The third is ND6 release into the bloodstream, which significantly increases in wildtype mice, but not in Pink1^EC-/-^ mice (new Figure 5E-F).

9) In Figure 6 A-B, does loss of PINK1 alter ND6 release in response to TNFa/FCCP?

We thank the reviewer for this important suggestion, addressing it has strengthened the paper. We addressed this by first determining whether the ND6 release phenotype occurred in vivo in response to LPS. Here, we collected plasma from mice injected with LPS and examined it for the presence of ND6 by immunoblotting. We found that ND6 is present in the plasma of LPS-injected mice, beginning at 6 hours post-LPS (new Figure 5E-F).

Next, to address the reviewer’s concern, we determined whether depleting endothelial Pink1 affected LPS-induced circulating ND6. Depletion of endothelial Pink1 significantly reduced LPS-mediated ND6 release (new Figure 5G-H). Thus, endothelial Pink1 is required for ND6 release.

10) Related to the mechanism of ND6 release, is it impaired by inhibition of lysosomal hydrolysis (for example with Bafilomycin A1)?

We thank the reviewer for this intriguing question. However, we believe that the mechanism of mitochondrial peptide release is outside the scope of the present study.

11) In Figure 6C-D, a non-formylated peptide control is also needed.

The activation of Erk in neutrophils downstream of fMLP and fMIT is well documented(Rane, Carrithers et al. 1997, Cao, Chen et al. 2023). The purpose of our experiment was to determine whether our dHL-60 cells were responsive to formyl peptides, using Erk signaling simply as a read-out. We do not want to claim that we are the first to show that formyl peptides activate Erk signaling. For the sake of clarity, this panel has been moved to a supplemental figure in the revised manuscript (Figure 5, figure supplement 1B-C).

12) In Figure 6E-G, ERK activation does necessarily mean an inflammatory response. Could the authors monitor some downstream markers such as cytokine release and if so, is this blocked by MEK/ERK inhibitors?

We appreciate the reviewer’s suggestion to study downstream effects. As our study highlights neutrophil recruitment, we examined neutrophil transmigration as a downstream effect of Erk activation. We employed a neutrophil transwell assay to evaluate whether conditioned media from endothelial cells treated with TNFα induced migration of dHL-60 cells. Compared to conditioned media from untreated cells, media from cells treated with TNFα induced significant transmigration of dHL-60s into the bottom chamber (new Figure 5A).

Reviewer #3 (Recommendations for the authors):

To establish this point, the authors need to demonstrate PINK1-dependent secretion of mitochondria-derived peptides after LPS addition in vivo. They also need to show an essential role for fMLP receptors on neutrophils in vivo. In its present state, the manuscript is inconclusive.

Technical criticisms

Regarding figure 1, as mitophagy is a very dynamic process and the Keima fluorophore is very sensitive to environmental conditions, I think that the authors should report the time required to prep and image these mouse lungs.

i.e. within 10 minutes of harvesting. This was possible due to the minimal processing required for imaging the lungs whole, without any sectioning. All images were taken within 1 hour of harvest. We have added this information to the methods section.

The quantification of in vivo mitophagy for figure 1 reports four animals per treatment condition, from what I assume was representative of three experiments. I struggle to understand how the authors could have normalized all PBS-treated datapoints to 1.0. It would be much better if the authors could report something simple with biological variation such as a mito-lysosomal area or intensity.

We thank the reviewer for bringing this to our notice. To clarify, lungs were analyzed in experimental pairs, consisting of PBS- LPS-injected from a single experiment, for a total of n=4 experiments. All microscope settings were kept consistent within one experiment. For each paired condition, the average of mitolysomsomal area of LPS-treated samples was normalized to that of PBS-treated sample on the same day. This information has been added to the figure methods section.

Similar to comment #2, the method of quantification for Figure 2B seems incorrect. The authors appear to be reporting variation in mitophagy between imaging fields. Please report the data across replicate experiments.

We thank the reviewer for identifying this error. We initially mistakenly believed that plotting the individual fields of view provided a better picture of the distribution of the %mitophagy across cells but agree with the reviewer that this was incorrect. The data has now been revised to show the average of all imaging fields for each experimental replicate (revised Figure 2B).

Regarding Figure 2c-d, the authors show an example of prolonged contact between a mitochondrion and a mito-lysosome. Without an untreated control and quantification, it is unconvincing that this is remarkable and/or unique to TNFa treatment.

In response, we have removed these figures from the manuscript.

Figure 5e, the authors need to state the timepoint used for this blot.

We apologize for this accidental omission. The examined time point was 6 hours following LPS treatment, and this has been added to the text in the Results section and in the figure legend (now moved to Figure 4F, G). We thank the reviewer for bringing this to our notice. We have also updated this data with additional replicates.

Figure 6 could also be supplemented with TEM to observe the ultrastructural of the TNFa-induced mitophagosomes, +/- PINK1 knockout.

We thank the reviewer for this intriguing suggestion. Although we would be interested in the results of this proposed experiment, we believe it to be outside the scope of this paper.

A number of conclusions are based on statistically underpowered figures. In Figure 4S1A and S2, and 5S1B, there are effects of similar or larger magnitude compared to other panels, but they do not reach significance because of small n.

We agree with the reviewer, and we have revised our conclusions to ensure that we do not over-interpret the data.

References:

Cao, Y., J. Chen, F. Liu, G. Qi, Y. Zhao, S. Xu, J. Wang, T. Zhu, Y. Zhang and Y. Jia (2023). "Formyl peptide receptor 2 activation by mitochondrial formyl peptides stimulates the neutrophil proinflammatory response via the ERK pathway and exacerbates ischemia-reperfusion injury." Cell Mol Biol Lett 28(1): 4.

Chen, K. H., L. M. Reece and J. F. Leary (1999). "Mitochondrial glutathione modulates TNF-α-induced endothelial cell dysfunction." Free Radic Biol Med 27(1-2): 100-109.

Chen, X., B. T. Andresen, M. Hill, J. Zhang, F. Booth and C. Zhang (2008). "Role of Reactive Oxygen Species in Tumor Necrosis Factor-α Induced Endothelial Dysfunction." Curr Hypertens Rev 4(4): 245255.

Drabarek, B., D. Dymkowska, J. Szczepanowska and K. Zabłocki (2012). "TNFα affects energy metabolism and stimulates biogenesis of mitochondria in EA.hy926 endothelial cells." Int J Biochem Cell Biol 44(9):1390-1397.

Forrester, S. J., K. J. Preston, H. A. Cooper, M. J. Boyer, K. M. Escoto, A. J. Poltronetti, K. J. Elliott, R. Kuroda, M. Miyao, H. Sesaki, T. Akiyama, Y. Kimura, V. Rizzo, R. Scalia and S. Eguchi (2020). "Mitochondrial Fission Mediates Endothelial Inflammation." Hypertension 76(1): 267-276.

Fu, P., Y. Epshtein, R. Ramchandran, J. B. Mascarenhas, A. E. Cress, J. Jacobson, J. G. N. Garcia and V. Natarajan (2021). "Essential role for paxillin tyrosine phosphorylation in LPS-induced mitochondrial fission, ROS generation and lung endothelial barrier loss." Sci Rep 11(1): 17546.

Grommes, J., M. Drechsler and O. Soehnlein (2014). "CCR5 and FPR1 mediate neutrophil recruitment in endotoxin-induced lung injury." J Innate Immun 6(1): 111-116.

Liang, W., S. Sagar, R. Ravindran, R. H. Najor, J. M. Quiles, L. Chi, R. Y. Diao, B. P. Woodall, L. J. Leon, E. Zumaya, J. Duran, D. M. Cauvi, A. De Maio, E. D. Adler and B. Gustafsson Å (2023). "Mitochondria are secreted in extracellular vesicles when lysosomal function is impaired." Nat Commun 14(1): 5031.

Rane, M. J., S. L. Carrithers, J. M. Arthur, J. B. Klein and K. R. McLeish (1997). "Formyl peptide receptors are coupled to multiple mitogen-activated protein kinase cascades by distinct signal transduction pathways: role in activation of reduced nicotinamide adenine dinucleotide oxidase." J Immunol 159(10):5070-5078.

Yang, S. C., S. H. Chang, P. W. Hsieh, Y. T. Huang, C. M. Ho, Y. F. Tsai and T. L. Hwang (2017). "Dipeptide HCH6-1 inhibits neutrophil activation and protects against acute lung injury by blocking FPR1." Free Radic Biol Med 106: 254-269.
